# Ninjurin1 drives lung tumor formation and progression by potentiating Wnt/β-Catenin signaling through Frizzled2-LRP6 assembly

**DOI:** 10.1186/s13046-022-02323-3

**Published:** 2022-04-08

**Authors:** Seung Yeob Hyun, Hye-Young Min, Ho Jin Lee, Jaebeom Cho, Hye-Jin Boo, Myungkyung Noh, Hyun-Ji Jang, Hyo-Jong Lee, Choon-Sik Park, Jong-Sook Park, Young Kee Shin, Ho-Young Lee

**Affiliations:** 1 Creative Research Initiative Center for concurrent control of emphysema and lung cancer, College of Pharmacy, Seoul National University, Seoul, 08826 Republic of Korea; 2 College of Pharmacy and Research Institute of Pharmaceutical Sciences, Seoul National University, Seoul, 08826 Republic of Korea; 3 School of Pharmacy, Sungkyunkwan University, Suwon-Si, Gyeonggi-do, 16419 Republic of Korea; 4 Soonchunhyang University Bucheon Hospital, Bucheon-si, Gyeonggi-do, 14584 Republic of Korea; 5 Department of Molecular Medicine and Biopharmaceutical Sciences, Graduate School of Convergence Science and Technology and College of Pharmacy, Seoul National University, Seoul, 08826 Republic of Korea

**Keywords:** Ninjurin1, cancer stem cell-like subpopulation, non-small cell lung cancer, Wnt signaling

## Abstract

**Background:**

Cancer stem-like cells (CSCs) play a pivotal role in lung tumor formation and progression. Nerve injury-induced protein 1 (Ninjurin1, Ninj1) has been implicated in lung cancer; however, the pathological role of Ninj1 in the context of lung tumorigenesis remains largely unknown.

**Methods:**

The role of Ninj1 in the survival of non-small cell lung cancer (NSCLC) CSCs within microenvironments exhibiting hazardous conditions was assessed by utilizing patient tissues and transgenic mouse models where Ninj1 repression and oncogenic *Kras*^G12D/+^ or carcinogen-induced genetic changes were induced in putative pulmonary stem cells (SCs). Additionally, NSCLC cell lines and primary cultures of patient-derived tumors, particularly Ninj1^high^ and Ninj1^low^ subpopulations and those with gain- or loss-of-*Ninj1* expression, and also publicly available data were all used to assess the role of Ninj1 in lung tumorigenesis.

**Results:**

Ninj1 expression is elevated in various human NSCLC cell lines and tumors, and elevated expression of this protein can serve as a biomarker for poor prognosis in patients with NSCLC. Elevated Ninj1 expression in pulmonary SCs with oncogenic changes promotes lung tumor growth in mice. Ninj1^high^ subpopulations within NSCLC cell lines, patient-derived tumors, and NSCLC cells with gain-of-*Ninj1* expression exhibited CSC-associated phenotypes and significantly enhanced survival capacities in vitro and in vivo in the presence of various cell death inducers. Mechanistically, Ninj1 forms an assembly with lipoprotein receptor-related protein 6 (LRP6) through its extracellular N-terminal domain and recruits Frizzled2 (FZD2) and various downstream signaling mediators, ultimately resulting in transcriptional upregulation of target genes of the LRP6/β-catenin signaling pathway.

**Conclusions:**

Ninj1 may act as a driver of lung tumor formation and progression by protecting NSCLC CSCs from hostile microenvironments through ligand-independent activation of LRP6/β-catenin signaling.

**Supplementary Information:**

The online version contains supplementary material available at 10.1186/s13046-022-02323-3.

## Background

Cancer poses a major threat to human health, and non-small cell lung cancer (NSCLC) is the leading cause of cancer-related deaths worldwide [1, 2]. Despite recent advances in diagnostic and treatment options, the 5-year survival rate for NSCLC remains relatively poor [3]. Cancer stem-like cells (CSCs), also known as tumor-initiating cells or tumor-propagating cells, are a rare subpopulation within the tumor and are defined by their capacity for self-renewal, anchorage-independence, and long-term clonal repopulation to generate primary, recurrent, and metastatic tumors with heterogeneity under various environmental insults [4, 5]. CSCs have been proposed to originate from the oncogenic transformation of normal stem/progenitor cells or due to dedifferentiation of genetically/epigenetically mutated transient amplifying or differentiated cells [4, 6]. In NSCLC, several cell surface enzymes such as CD133, CD44, CD166, EpCAM, and aldehyde dehydrogenase 1 (ALDH1) have been suggested as putative CSC-associated markers [7]. In particular, CSC marker expression was associated with poor clinical outcomes in patients with NSCLC, particularly those with lung adenocarcinoma (ADC) [8]. Therefore, the successful targeting of CSCs may provide an innovative approach for eradicating tumors. However, the molecular mechanisms underlying the phenotypic and functional features of NSCLC CSCs remain unclear.

The Wnt/β-catenin signaling pathway has been implicated in the proliferation, motility, and maintenance of stem cells (SCs) to thereby contribute to regeneration [9, 10]. The Wnt/β-catenin signaling pathway has been demonstrated to engage in crosstalk with various pro-survival pathways such as the phosphoinositide 3-kinase/Akt, mitogen-activated protein kinase, and signal transducer and activator of transcription signaling pathways, thus leading to resistance to apoptotic stimuli [11]. In the absence of ligands, a cytosolic destruction complex composed of Axin, adenomatous polyposis coli (APC), glycogen synthase kinase-3β (GSK-3β), and casein kinase I (CKI) mediates the phosphorylation and proteasomal degradation of β-catenin [9]. Upon Wnt binding to the frizzled (FZD) and lipoprotein receptor-related protein (LRP) dual-receptor complex, β-catenin is released from the multiprotein destruction complex [9], thus resulting in its nuclear translocation and the expression of various genes involved in the maintenance of tissue-specific SCs and cell survival [9, 10]. Despite its role in tissue homeostasis, aberrant stimulation of the Wnt/β-catenin pathway through mutational loss of *APC* or stabilization of β-catenin promotes tumor formation and progression [12–14]. However, the frequency of these mutations is low in various cancer types, including lung cancer [15, 16], and very little is known regarding the mechanisms that control Wnt/β-catenin signaling in these cancers.

Nerve injury-induced protein 1 (Ninjurin1; hereafter Ninj1) is a 17-kDa homophilic cell adhesion molecule located in the cell membrane that is composed of an N-terminal extracellular domain, a cytosolic domain, two transmembrane domains, and a C-terminal domain [17]. Ninj1 has been identified as a protein that is induced by nerve injury to mediate cell adhesion and neuronal regeneration [17, 18]. Post-translational modification of Ninj1 occurs through glycosylation, and this is one of the characteristics of the cell surface proteins of CSCs [19]. Ninj1 expression is induced in response to various stresses within the tumor microenvironment (TME) [17, 20–22] and plays an important role in macrophage-mediated inflammation and vascular remodeling [23, 24], both of which have been closely implicated in cancer development and progression [25]. Ninj1 has been proposed to play a dual role in lung tumorigenesis depending on the p53 mutation status [26]. Additionally, Ninj1 is overexpressed in various cancers, including hepatocellular carcinoma [27], acute lymphoblastic B-cell leukemia [28], urothelial bladder cancer [29], and circulating prostate cancer cells [30]. However, the role of Ninj1 in neoplasia is controversial, as it is known to exhibit both tumor-promoting and tumor-inhibiting activities, and its mechanism of action is largely unknown [21, 26, 31].

In the present study, we aimed to understand the pathological role of Ninj1 in the context of NSCLC as a basis for developing CSC-targeting therapeutic strategies for patients with NSCLC. Our results demonstrate that Ninj1 renders NSCLC CSCs resistant to apoptotic stimuli from the microenvironment by activating the Wnt/β-catenin signaling pathway through assembly with LRP6 and FZD2. Our results suggest that Ninj1 is a potential target for anti-CSC strategies to suppress tumor growth and overcome anticancer drug resistance in patients with NSCLC.

## Methods

Additional experimental procedures are described in the Supplementary Methods.

### Cell culture

Human NSCLC cell lines (H1299, H460, A549, H1975, H292, H522, and HCC827) were purchased from the American Type Culture Collection (ATCC, Manassas, VA, USA). Other human NSCLC cells (Calu-1, H1944, H226B, H226Br, HCC-15, and PC-9) were kindly provided by Dr. John V. Heymach (MD Anderson Cancer Center, Houston, TX, USA). These cells were cultured in RPMI 1640 supplemented with 10% fetal bovine serum (FBS) and antibiotics (Welgene, Kyeongsan-si, Republic of Korea). Genetic alterations in these NSCLC cell lines are presented in Table S1. HB56B and BEAS-2B cells were kindly provided by Dr. R. Reddel (National Cancer Institute, Bethesda, MD, USA) and Dr. A. Klein-Szanto (Fox Chase Cancer Center, Philadelphia, PA, USA), respectively. HBE cells were kindly provided by Dr. John D. Minna (The University of Texas Southwestern Medical Center, Dallas, Texas, USA). These normal cells were cultured in K-SFM (Invitrogen, Grand Island, NY, USA) supplemented with 5 ng/mL of recombinant epidermal growth factor (EGF), 50 mg/mL of bovine pituitary extracts, and antibiotics. NSCLC cell lines were authenticated and validated using the AmplFLSTR identifier PCR Amplification Kit (Applied Biosystems, Foster, CA; cat. No. 4322288) in 2013, 2016, and 2020. Cells cultured for fewer than 3 months after resuscitation of validated cells and that were confirmed to be mycoplasma-free were used in this study.

### Reagents

Mouse monoclonal anti-human Ninj1 primary antibodies that were used for western blot analysis were purchased from R&D Systems (Minneapolis, MN, USA). The human monoclonal anti-Ninj1 primary antibodies used for immunofluorescence staining and flow cytometry were kindly provided by Dr. Young Kee Shin (Seoul National University, Seoul, Republic of Korea). Rabbit polyclonal anti-mouse Ninj1 primary antibody used for immunohistochemistry was kindly provided by Dr. Kyu-Won Kim (Seoul National University, Seoul, Republic of Korea) [1]. Tissue microarrays were purchased from US Biomax (Rockville, MD, USA). Antibodies specific for pLRP6 (S1490), LRP6, FZD2, Dvl3, Axin1, GSK-3β, Nanog, cleaved caspase-3, and tubulin were purchased from Cell Signaling Technology (Danvers, MA, USA). Antibodies specific for cleaved poly ADP-ribose polymerase (PARP), and Matrigel were purchased from BD Biosciences (San Jose, CA, USA). Primary antibodies targeting β-catenin, 6x-His tag, GST, PARP, and actin were purchased from Santa Cruz Biotechnology (Dallas, TX, USA). Antibodies specific for Oct4 and Sox2 were purchased from Abcam (Cambridge, UK). Horseradish peroxidase (HRP)-conjugated secondary antibodies were purchased from GeneTex (Irvine, CA, USA). Ni-NTA agarose and fluorescent (Alexa Fluor 488 and Alexa Fluor 594)-conjugated secondary antibodies were purchased from Thermo Fisher Scientific (Waltham, MA, USA). Fluorescein isothiocyanate (FITC)-conjugated anti-human secondary antibodies were purchased from Jackson ImmunoResearch Laboratories (West Grove, PA, USA). The 3-(4,5-dimethylthiazol-2-yl)-2,5-diphenyl tetrazolium bromide (MTT) reagent was purchased from MP Biomedicals (Santa Ana, CA, USA). The ALDH Detection Assay Kit and anti-active β-catenin (β-catenin^act^) primary antibody were purchased from Merck Millipore (Billerica, MA, USA). 4-(methylnitrosamino)-1-(3-pyridyl)-1-butanone (NNK) was purchased from Toronto Research Chemicals Inc. (North York, Ontario, Canada). Propidium iodide (PI), benzo[a]pyrene (B[a]P), tamoxifen, urethane, corn oil, bovine serum albumin (BSA), EDTA, and other chemicals were purchased from Sigma-Aldrich (St. Louis, MO, USA) unless otherwise specified. Detailed information regarding the primary and secondary antibodies used, including vendor, catalogue number, application, and dilution ratio (or concentration), is listed in Table S2.

### Animal experiments

All animal procedures were performed according to protocols approved by the Seoul National University Institutional Animal Care and Use Committee. Mice were freely provided with standard chow and water and housed in a temperature- and humidity-controlled facility under a 12-h light/12-h dark cycle.

Animal experiments were performed as described previously [32, 33]. To compare Ninj1 expression in the cancerous region of the lungs to the normal or tumor-adjacent normal regions, the lungs from five-month-old male and female *Kras*^G12D/+^ transgenic mice or from male and female FVB/N mice treated with 3 μmol NNK and 3 μmol B[a]P dissolved in corn oil and administered by oral gavage for 5 months were analyzed. For the xenograft experiment, NSCLC cells (1 × 10^6^ cells/spot, diluted in equal amounts of PBS and Matrigel) or patient-derived tumors were subcutaneously inoculated into the right flank of 6-week-old female NOD/SCID mice. After the tumor volume reached 50–100 mm^3^, the mice were randomly grouped and intraperitoneally treated with either vehicle or a combination of paclitaxel (20 mg/kg) that was dissolved in a mixture of Cremophor EL and ethanol (1:1, v/v) and further diluted in PBS (final 1:1:18, v/v/v)] and cisplatin (3 mg/kg) that was dissolved in 0.9% (w/v) NaCl solution for once a week [33]. Tumor growth was determined by measuring the short and long diameters of the tumor using a caliper, and tumor volume was calculated using the formula: tumor volume (mm^3^) = (small diameter)^2^ × (large diameter) × 0.5 [32, 33]..

Detailed information regarding the conditional Ninj1 transgenic mouse (LSL-*Ninj1*^Tg/+^) was previously described in our recent study [31]. *Scgb1a1*-CreER™ and *Sftpc*-CreER^T2^ mice with a C57BL/6 J background were kindly provided by Dr. Brigid Hogan (Duke University, Durham, NC, USA). These mice were backcrossed onto an FVB/N background with FVB/N mice (purchased from Japan SLC, Inc., Hamamatsu, Japan) for more than eight generations. To induce the Ninj1 transgene, 3-week-old *Sftpc*-CreER^T2^;LSL-*Ninj1*^Tg/+^;*Kras*^G12D/+^ and *Scgb1a1*-CreER^TM^;LSL-*Ninj1*^Tg/+^ mice were intraperitoneally administered with vehicle (corn oil) or 0.25 mg/g body weight of tamoxifen (dissolved in corn oil) once a day for 5 consecutive days. For the *Scgb1a1*-CreER^TM^;LSL-*Ninj1*^Tg/+^ mice, 1 g/kg of urethane was intraperitoneally administered to facilitate tumor formation. After 12 months, bioluminescence images were obtained using IVIS-Spectrum microCT and Living Images (ver. 4.2) software (PerkinElmer, Alameda, CA, USA) using an MMPSense 680 probe (PerkinElmer; 2 nmol/150 μl in PBS) according to the manufacturer’s instructions. The mice were euthanized, and a postmortem examination was performed to evaluate tumor formation in the lungs. To measure the mean tumor number (N) and volume (V), H&E-stained lung tissues were observed under a microscope in a blinded manner. The number and sizes of the tumors were calculated in five sections that were uniformly distributed throughout each lung. The tumor volume and burden of each sample were calculated using the formula: tumor volume (mm^3^) = (short diameter)^2^ × (long diameter) × 0.5; tumor burden = number of tumors × the average tumor volume [32].

### Isolation of the Ninj1^high^ and Ninj1^low^ populations

H460 and A549 cells or primary lung tumor cells derived from PDXs were stained with anti-Ninj1 antibodies were diluted in FACS buffer (PBS containing 1% BSA, 2 mM EDTA, and 0.05% sodium azide; 1:100 ratio), washed twice with FACS buffer, and stained with FITC-conjugated secondary antibodies. After washing twice with FACS buffer, the stained cells were sorted using a FACS Aria III flow cytometer (BD Biosciences) for further in vitro experiments.

### Statistics

Data are presented as mean ± SD. All in vitro experiments were independently performed at least three times, and the representative results are presented. The data were analyzed using GraphPad Prism software (version 9, GraphPad Software, San Diego, CA, USA). Statistical significance was determined using two-tailed Student’s t-test, Mann-Whitney test, one-way analysis of variance (ANOVA), or Brown-Forsythe and Welch ANOVA tests. An F-test for equality of variances was performed to ensure the same variance between the two test groups. The Brown–Forsythe test for equality of variances was performed to ensure the same variance in more than three experimental groups. The Shapiro-Wilk test was performed to determine if the in vitro or in vivo data followed a normal distribution. Statistical significance was set at *P* < 0.05.

## Results

### Elevated Ninj1 expression promotes lung tumorigenesis

**Fig. 1:**
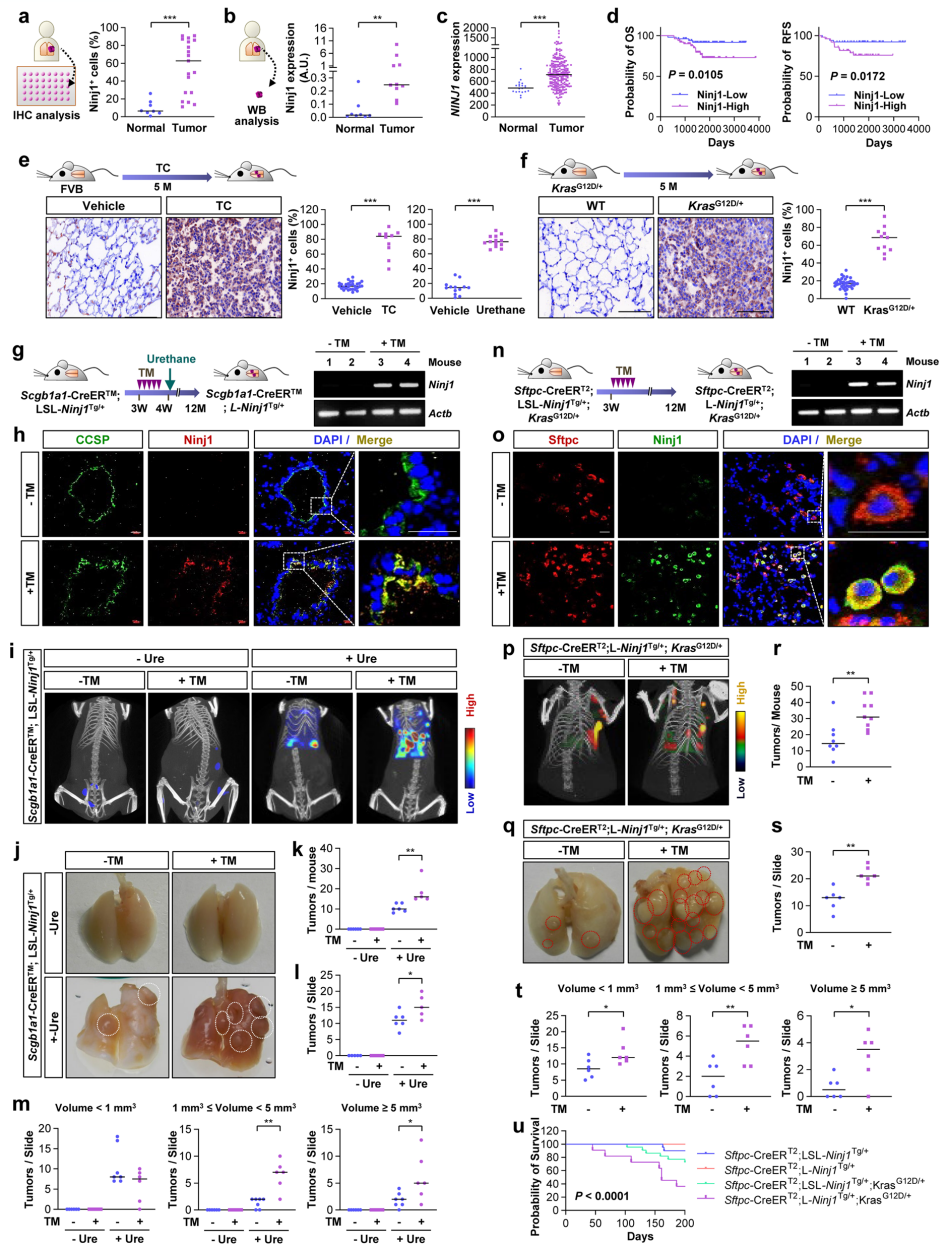
Ninj1 promotes lung tumor development and progression. **a** Immunohistochemical (IHC) analysis of Ninjurin1 (Ninj1) expression in a tissue microarray of patient-derived normal lung and tumor tissues. **b** Western blot analysis of Ninj1 expression in patient-derived normal lung and tumor tissues. The densitometry of each Ninj1 blot was analyzed using the ImageJ software. **c, d** Analysis of a GEO dataset (GSE31210) for (**c**) *NINJ1* expression in lung tumors in patients with NSCLC and (**d**) the Kaplan–Meier estimates for the association of *NINJ1* expression with overall (OS) and relapse-free (RFS) survival in patients with NSCLC. The *P* value was determined according to the log-rank test. The top and bottom 25th percentiles were used to determine the Ninj1^high^ and Ninj1^low^ groups, respectively. **e, f** IHC analysis of Ninj1 expression in normal lung tissues from control mice and lung tumors from mice treated with tobacco carcinogen (TC)- (**e**) or urethane (**e**) or harboring the *Kras* mutations (*Kras*^G12D/+^; **f**). The number of Ninj1^+^ cells was determined using ImageJ software. **g, n** Schematic diagram for the experimental schedule in *Scgb1a1*-CreER^TM^;LSL-*Ninj1*^Tg/+^ mice (**g**) and *Sftpc*-CreER^T2^;LSL-Ninj1^Tg/+^;*Kras*^G12D/+^ mice (**n**). These mice were treated with vehicle (corn oil) or tamoxifen (TM) for five consecutive days at 3 weeks of age. *Scgb1a1*-CreER^TM^; L-*Ninj1*^Tg/+^ *-* mice were further exposed to urethane (Ure) at 4 weeks of age. RT-PCR analysis examining Ninj1 mRNA expression in the lungs (**g, n**), and immunofluorescence analysis examining Ninj1 protein expression (**h, o**) in Scgb1a1^+^ (CCSP^+^) (**h**) or Sftpc^+^ (**o**) lung epithelial cells. Scale bars: 20 μm. Lung tumor formation was analyzed using IVIS images (**i, p**), gross observation (**j, k, q, r**), and microscopic analysis of the H&E-staining of the lung tissues (**l, m, s, t**) in *Scgb1a1*-CreER^TM^;LSL-*Ninj1*^Tg/+^ mice (**i-m**) and *Sftpc*-CreER^T2^;LSL-*Ninj1*^Tg/+^;*Kras* ^G12D/+^ mice (**p-t**). Numeration of lung tumors through gross (**k, r**) and microscopic evaluation (**l, m, s, t**). **u** The Kaplan–Meier estimates for the survival of mice carrying *Sftpc*-CreER^T2^;LSL-*Ninj1*^Tg/+^ and of *Sftpc*-CreER^T2^;LSL-*Ninj1*^Tg/+^;*Kras*^G12D/+^ mice treated with vehicle or tamoxifen. The *P* value was determined using the log-rank test. The bars represent the mean ± SD; *P < 0.05, **P < 0.01, and ***P < 0.001, as determined by a two-tailed Student’s *t*-test or Mann-Whitney test by comparison to the indicated group or by one-way ANOVA with Tukey’s post-hoc test (**k-m**). WT: wild-type

To investigate the possible role of Ninj1 in the context of lung tumors, we performed immunohistochemical (IHC) analysis of Ninj1 expression in a tissue microarray composed of NSCLC tissues (*n* = 40) and normal lung tissues (*n* = 10). Significantly greater Ninj1 staining was observed in NSCLC tissues compared to that in normal lung tissues (*P* < 0.001) (Fig. 1a). Western blot analysis of tissue samples from the certified human bioresource bank in Korea [34] confirmed significantly elevated Ninj1 protein expression in lung tumor tissues (*n* = 10) compared to that in normal lung tissues (*n* = 8) (*P* = 0.0014) (Fig. 1b). Analyses of publicly available datasets from NSCLC patients further revealed that *NINJ1* mRNA expression was significantly elevated in tumor tissues compared to levels in normal tissues (*P* < 0.001) (Fig. 1c) and was associated with poor overall and relapse-free survival in NSCLC patients (OS: *P* = 0.0105; RFS: *P* = 0.0172) (Fig. 1d). Next, we analyzed Ninj1 expression in mice harboring lung tumors caused by exposure to carcinogens such as urethane or the combination of 4-(methylnitrosamino)-1-(3-pyridyl)-1-butanone and benzo[a]pyrene (tobacco carcinogens, TC) [35, 36] (Fig. 1e, S1a) or caused by the oncogenic *Kras* mutation (*Kras*^G12D/+^) that is an established a genetic alteration that is characteristic of lung cancer [37](Fig. 1f). Consistent upregulation of Ninj1 expression was observed in these tumors compared to levels in normal lung tissues in control mice (*P* < 0.001).

To﻿ investigate the functional role of Ninj1 in lung tumorigenesis, we created a conditional transgenic (Tg) mouse model termed LoxP-stop-LoxP (LSL)-*Ninj1*^Tg/+^ and then crossed it with mice harboring the *Scgb1a1*-CreER^TM^ recombinase-estrogen receptor fusion protein transgene [38] (Fig. S1b). We treated LSL-*Scgb1a1*-CreER^TM^;LSL-*Ninj1*^Tg/+^ mice with five doses of tamoxifen (TM) at 3 weeks of age and confirmed the presence of increased *Ninj1* mRNA levels in the lungs through the use of PCR analysis (Fig. 1g). Immunofluorescence (IF) staining of lung tissues further revealed that approximately 80% of CCSP^+^ cells were Ninj1^+^ in *Scgb1a1*-CreER^TM^;L-*Ninj1*^Tg/+^ mice (Fig. 1h). No tumor nodules were observed in *Scgb1a1*-CreER^TM^;L-*Ninj1*^Tg/+^ mice at up to 1 year of age. Lung tumor initiation was induced by exposing *Scgb1a1*-CreER^TM^;L-*Ninj1*^Tg/+^ mice to urethane at 4 weeks of age. Bioluminescence imaging (Fig. 1i) and gross evaluation of the lung (Fig. 1j, k) revealed significantly more lung tumor nodules in *Scgb1a1*-CreER^TM^;L-*Ninj1*^Tg/+^ mice compared to that in *Scgb1a1*-CreER^TM^;LSL-*Ninj1*^Tg/+^ mice following exposure to urethane (*P* = 0.0011). Microscopic analyses revealed that the number of tumor nodules (*P* = 0.0259) (Fig. 1l), particularly those larger than 1 mm^3^ (Fig. 1m), was significantly higher in *Scgb1a1*-CreER^TM^;L-*Ninj1*^Tg/+^ mice than it was in *Scgb1a1*-CreER^TM^;LSL-*Ninj1*^Tg/+^ mice (1 mm^3^ ≤ volume < 5 mm^3^: *P* < 0.001; volume ≥ 5 mm^3^: *P* = 0.0154).

Next, LSL-*Ninj1*^Tg/+^;*Sftpc*-CreER^T2^;*Kras*^G12D/+^ mice were established by crossing LSL-*Ninj1*^Tg/+^;*Sftpc*-CreER^T2^ mice with *Kras*^G12D/+^ Tg mice. TM exposure-induced *Sftpc*-CreER^T2^;LSL-*Ninj1*^Tg/+^;*Kras*^G12D/+^ mice exhibited increased *Ninj1* mRNA levels in the lung (Fig. 1n) and elevated Ninj1 protein expression in approximately 80% of SPC^+^ alveolar type II epithelial cells (AT2s) (Fig. 1o). *Sftpc*-CreER^T2^;L-*Ninj1*^Tg/+^;*Kras*^G12D/+^ mice exhibited significantly increased lung tumor formation compared to that in *Sftpc*-CreER^T2^;LSL-*Ninj1*^Tg/+^;*Kras*^G12D/+^ mice according to bioluminescence imaging (Fig. 1p) and gross analysis of lung tumor nodules (*P* = 0.0056) (Fig. 1q, r). Microscopic analyses revealed that *Sftpc*-CreER^T2^;L-*Ninj1*^Tg/+^;*Kras*^G12D/+^ mice possessed significantly higher amounts of tumor nodules (*P* = 0.001) (Fig. 1s), particularly those larger than 1 mm^3^ (volume < 1 mm^3^: *p* = 0.0406; 1 mm^3^ ≤ volume < 5 mm^3^: *P* = 0.0088; volume ≥ 5 mm^3^, *P* = 0.0157) (Fig. 1t), and exhibited worse overall survival (*P* < 0.001) (Fig. 1u) compared to these characteristics in control mice. These results collectively indicate the role of Ninj1 as a driver of lung tumorigenesis and suggest that this protein may serve as a clinically useful biomarker for poor prognosis in patients with NSCLC.

### Ninj1 induces CSC phenotypes and survival potential against diverse cell death inducers, thus promoting lung tumor formation

**Fig. 2:**
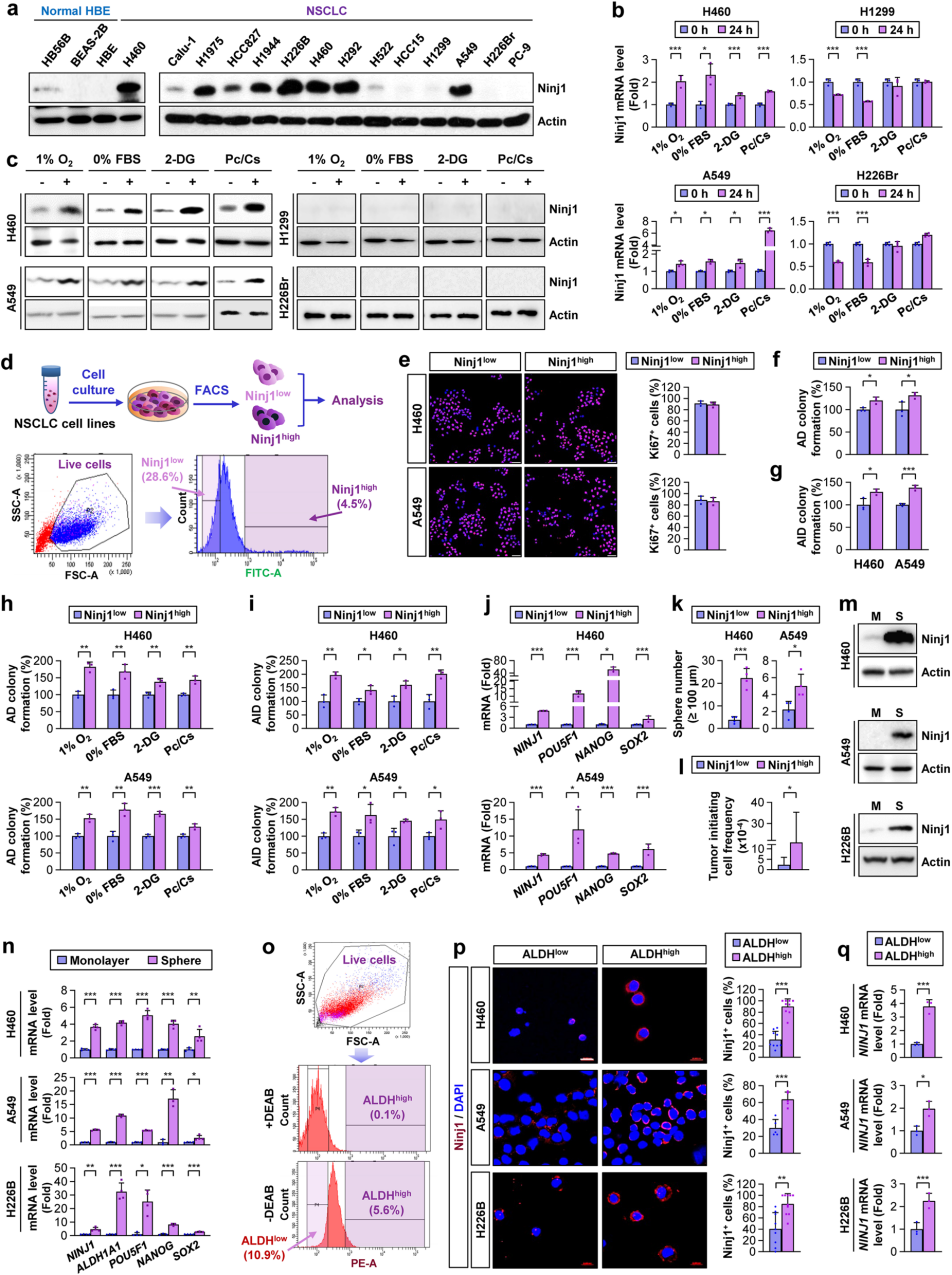
Ninj1 is tightly associated with the survival capacity and CSC-like traits of NSCLC cells. **a** Western blot analysis of Ninjurin1 (Ninj1) expression in the indicated NSCLC and normal HBE cell lines. **b, c** Real-time PCR (**b**) and Western blot (**c**) analyses of the levels of Ninj1 mRNA and protein expression, respectively, under culture conditions of hypoxia (1% O_2_), serum starvation (0% FBS), glucose deprivation (1 mM 2-deoxy-L-glucose, 2-DG), or chemotherapy treatment (10 nM paclitaxel and 10 μM cisplatin in combination; Pc/Cs). **d** Schematic diagram and gating strategy of flow cytometry sorting for Ninj1^low^ and Ninj1^high^ populations. **e-g, j, k, l** The basal Ki67 positivity (**e**) and the anchorage-dependent (AD) (**f**) and -independent (AID) colony formation (**g**), mRNA expression of *NINJ1* and CSC markers (*POU5F1, NANOG,* and *SOX2*) (**j**), sphere formation (**k**), and tumorigenicity (l) of Ninj1^low^ and Ninj1^high^ subpopulation of H460 (**e-g, j, k**) and A549 (**e-g, j, k, l**) cells. Scale bars: 50 μm (**e**). Tumor initiating cell frequency was determined according to ELDA (**l**). **h, i** Anchorage-dependent (AD) (**h**) and -independent (AID) colony formation (**i**) of Ninj1^high^ cells under hypoxia (1% O_2_), serum starvation, glucose deprivation, and exposure to chemotherapy (Pc/Cs) compared to those of Ninj1^low^ cells. **m, n** Western blot (**m**) and/or real-time PCR (**n**) analyses of the levels of Ninj1 protein and mRNA expression and also of the CSC marker genes in the indicated NSCLC cells grown in monolayer (M) or sphere-forming conditions (S). **o** Gating strategy to isolate ALDH^high^ and ALDH^low^ populations. **p, q** Immunofluorescence (**p**) and real-time PCR (**q**) analyses assessing the levels of Ninj1 protein and mRNA expression, respectively, in the ALDH^low^ and ALDH^high^ populations in the indicated NSCLC cell lines. The Ninj1^+^ cells were determined using ZEN software. Scale bar: 20 μm (**o**). All experiments were performed at least three times. The bars represent the mean ± SD; *P < 0.05, **P < 0.01, and ***P < 0.001, as determined by a two-tailed Student’s *t*-test or Mann-Whitney test by comparison to the indicated group

We investigated mechanism by which Ninj1 promotes the growth of lung tumors. As *NINJ1* has been reported as a p53 target gene [21], we analyzed Ninj1 expression in normal human bronchial epithelial (NHBE) cell lines (HB56B, BEAS-2B, and HBE), 13 human NSCLC cell lines carrying wild-type (WT) *TP53* that encodes the p53 protein (H1944, H226B, H460, H292, and A549), or mutant/null *TP53* cell lines (Calu-1, H1975, HCC827, H522, HCC15, H1299, H226Br, and PC-9) (Table S1). As anticipated, most NSCLC cell lines possessed higher levels of Ninj1 expression compared to that in the three NHBE cell lines (Fig. 2a). However, neither *TP53* mutations nor *KRAS* or epidermal growth factor receptor (*EGFR*) mutations exhibited an obvious correlation with basal levels of Ninj1 expression in these NSCLC cells (Table S1). As diverse pyroptotic, necrotic, and apoptotic cell death inducers have been demonstrated to induce p53-dependent increases in Ninj1 expression in macrophages [22], we next analyzed the response of NSCLC cell lines carrying WT or mutant/null *TP53* to pyroptotic (i.e., hypoxia, paclitaxel, and cisplatin) [39, 40], necrotic (i.e., hypoxia, glucose deprivation, paclitaxel, and cisplatin) [41–44], and apoptotic (i.e., hypoxia, serum or glucose deprivation, paclitaxel, and cisplatin) stimuli [41, 45]. Indeed, NSCLC cell lines carrying WT *TP53* (H460 and A549) and not those carrying null/mutant *TP53* (H1299 and H226Br) exhibited marked increases in the mRNA (Fig. 2b) and protein expression (Fig. 2c) of Ninj1.

We also investigated how Ninj1 promotes the growth of lung tumors by employing Ninj1^high^ and Ninj1^low^ NSCLC cell subpopulations that were sorted using flow cytometry (Fig. 2d). Compared to the Ninj1^low^ subpopulations (lower 28.6% of live cells) from H460 and A549 cells, Ninj1^high^ cells (upper 4.5% of live cells) derived from the corresponding parental cells exhibited similar Ki67 expression (Fig. 2e). In contrast, the results from a clonogenic assay (i.e., an anchorage-dependent colony formation assay that evaluates the survival of a single cell and proliferation into a colony) [46] (Fig. 2f, h) and from a soft-agar colony formation assay (an anchorage-independent formation assay that evaluates the survival and proliferation of cells within a harsh environment under unattached conditions) [47] (Fig. 2g, i) revealed more prominent colony-forming capacities in the Ninj1^high^ subpopulations than were observed in the Ninj1^low^ subpopulations when cultured under normal culture conditions (Fig. 2f, g) or in the presence of diverse cell death inducers (Fig. 2h, i).

Given the traits of CSCs in regard to resistance to hazardous microenvironments [4], we hypothesized that Ninj1 endows NSCLC cells with CSC phenotypes, including survival capacity against hazardous environments. Ninj1^high^ subpopulations in H460 and A549 cells exhibited increased CSC properties, including CSC-associated marker gene expression (i.e., *ALDH1A1*, *POU5F1*, *NANOG*, and *SOX2*) (Fig. 2j), tumorsphere formation [8] (Fig. 2k), and tumorigenicity in the limiting dilution assay (*P* = 0.0171) (Fig. 2l) compared to these characteristics the Ninj1^low^ subpopulations and their corresponding NSCLC cells. The H460 and A549 subpopulations obtained from sphere-forming culture conditions also possessed increased Ninj1 expression (Fig. 2m) and CSC-associated marker gene expression (Fig. 2n) compared to that of their corresponding NSCLC cells cultured under monolayer conditions. Moreover, subpopulations from H460, A549, and H226B cells were obtained by increasing the activity of aldehyde dehydrogenase (ALDH) [48] (Fig. 2o), another general property of CSCs, and these cells consistently possessed upregulated Ninj1 protein (Fig. 2p) and mRNA (Fig. 2q) expression levels of Ninj1 compared to levels in ALDH^low^ subpopulations and their corresponding NSCLC cells.

**Fig. 3:**
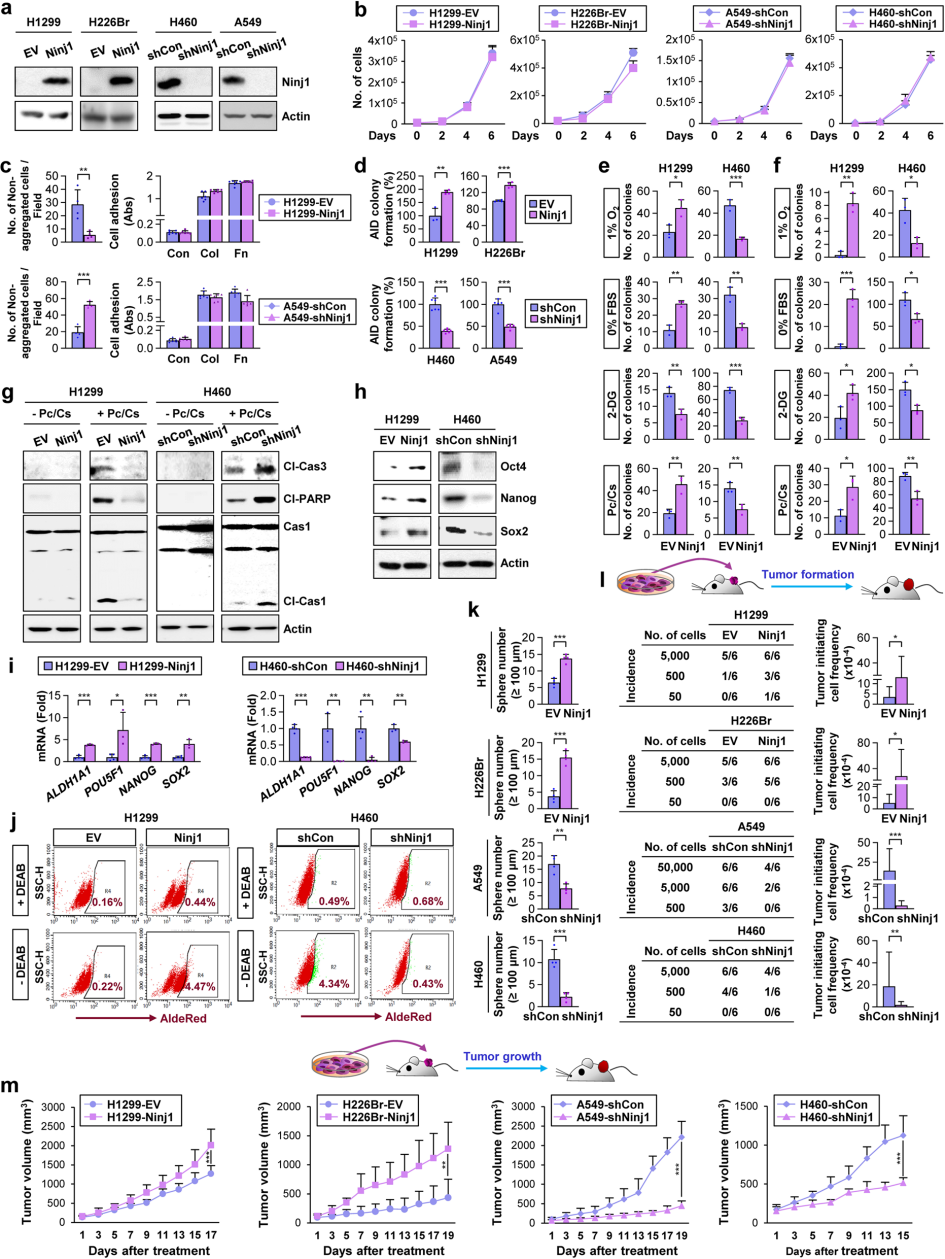
Ninj1 mediates the acquisition of CSC phenotypes in NSCLC cell lines. **a** Western blot analysis examining Ninjurin1 (Ninj1) expression in the indicated NSCLC cells with an enforced or knocked down Ninj1 expression via stable transfection with a mammalian expression vector or shRNA, respectively. **b-d** NSCLC cells that achieved upregulation (H1299-Ninj1 and H226Br-Ninj1) or downregulation (A549-shNinj1 and H460-shNinj1) of Ninj1 were subjected to cell counting (**b**), hanging drop assays for cell-to-cell cohesion (**c; left**), cell adhesion assays for cell adhesion to the extracellular matrix (type I collagen [Col] and fibronectin [Fn]) adhesion (**c; right**), and anchorage-independent (AID) colony-forming assays (**d**). **e-l** Analyses of the indicated NSCLC cell lines with manipulation of Ninj1 expression via anchorage-dependent (AD) (**e**) and -independent (AID) (**f**) colony formation under hypoxia (1% O_2_), serum starvation (0% FBS), glucose deprivation (1 mM 2-deoxy-L-glucose [2-DG]), and exposure to paclitaxel (10 nM) and cisplatin (10 μM) in combination (Pc/Cs); Western blot analysis revealing pyroptotic or apoptotic cell death by treatment with Pc/Cs (**g**); Western blot (**h**) and real-time PCR (**i**) analyses on the levels of protein and mRNA expression of CSC markers; flow cytometric ALDH assay (**j**); sphere formation analysis (**k**); limiting dilution assay examining tumorigenic potential (**l**). Tumor initiating cell frequency was determined using ELDA (**l**). **m** Growth of xenograft tumors from the indicated NSCLC cell lines with stable overexpression/or knockdown of Ninj1 expression. All in vitro experiments were performed at least three times. The bars represent the mean ± SD; *P < 0.05, **P < 0.01, and ***P < 0.001, as determined by a two-tailed Student’s *t*-test or Mann-Whitney test by comparison to the indicated group. Cl-Cas3: cleaved caspase 3; Cl-Cas1: cleaved caspase 1; Cas1: caspase 1

To provide direct evidence for the functional role of Ninj1 in CSC phenotypes and tumorigenic activities in NSCLC cells, we selected Ninj1^low^ (H1299 and H226Br cells) and Ninj1^high^ (A549 and H460) expression cells and established their sublines that were stably transfected with an expression vector that was empty (EV) or carrying either human Ninj1 or control or Ninj1-specific shRNA, respectively. The established cells that exhibited forced overexpression (H1299-Ninj1 and H226Br-Ninj1) or downregulation (A549-shNinj1 and H460-shNinj1) of Ninj1 expression and their corresponding control cells (H1299-EV, H226Br-EV, A549-shCon, or H460-shCon)(Fig. 3a) possessed similar proliferation rates (Fig. 3b). In agreement with the role of Ninj1 as a cohesion molecule [17], the established cells possessing upregulation or downregulation of Ninj1 expression exhibited significantly increased or decreased cell-cell cohesion, respectively, without any detectable changes in their adhesion to extracellular matrix (ECM) components such as type I collagen (Col) and fibronectin (Fn) (Fig. 3c).

When cultured under normal conditions, H1299-Ninj1 and H226Br-Ninj1 cells exhibited significantly greater capacities for colony formation, while A549-shNinj1 and H460-shNinj1 cells possessed decreased colony formation capacities compared to those of the corresponding control cells (Fig. 3d). When exposed to the diverse cell death inducers described above, H1299-Ninj1 cells exhibited significantly increased anchorage-dependent (Fig. 3e) and anchorage-independent (Fig. 3f) colony-forming capacity and decreased caspase 1 and caspase 3 cleavage (markers for pyroptotic or apoptotic cell death, respectively [49]) (Fig. 3g) compared to that of their control cells. Conversely, resistance to these cell death inducers was significantly decreased in H460-shNinj1 cells compared to that in H460-shCon cells (Fig. 3e-g). Furthermore, compared to their control cells, CSC-associated phenotypes, including protein (Fig. 3h) and mRNA (Fig. 3i) expression of CSC markers and ALDH activity (Fig. 3j), were higher in H1299-Ninj1 cells and attenuated in H460-shNinj1 cells. Consistently, sphere-forming activities were significantly increased by Ninj1 expression and were attenuated by Ninj1 silencing **(**Fig. 3k).

Next, we analyzed the tumorigenic capacity of these established NSCLC cells using an in vivo limiting dilution assay. H1299-Ninj1 and H226Br-Ninj1 cells possessed significantly greater tumorigenicity than did their corresponding control cells, while A549-shNinj1 and H460-shNinj1 cells exhibited significantly decreased tumorigenicity (Fig. 3l). Once developed, xenograft tumors from H1299-Ninj1 and H226Br-Ninj1 cells displayed significantly faster growth than did their control tumors, while those from A549-shNinj1 and H460-shNinj1 cells exhibited significantly slower growth compared to that of their control tumors (Fig. 3m). The expression of CSC markers (Oct4 and Nanog) was increased in H1299-Ninj1 xenograft tumors and was attenuated in H460-shNinj1 xenograft tumors compared to that in their corresponding control tumors (Fig. S2a). An associated elevation in Ninj1 and Nanog expression was also observed in tumor nodules in *Scgb1a1*-CreER^TM^;L-*Ninj1*^Tg/+^ and *Sftpc*-CreER^T2^;L-*Ninj1*^Tg/+^;*Kras*^G12D/+^ mice (Fig. S2b). These findings suggest that Ninj1 expression that is increased either through innate mechanisms or through the action of cell death inducers protects NSCLC cells from various environmental insults in the tumor, thus promoting tumor development and growth.

### Ninj1^high^ subpopulations in human NSCLC exhibit increased CSC traits and survival potential against pyroptotic, necrotic and apoptotic cell death inducers

**Fig. 4:**
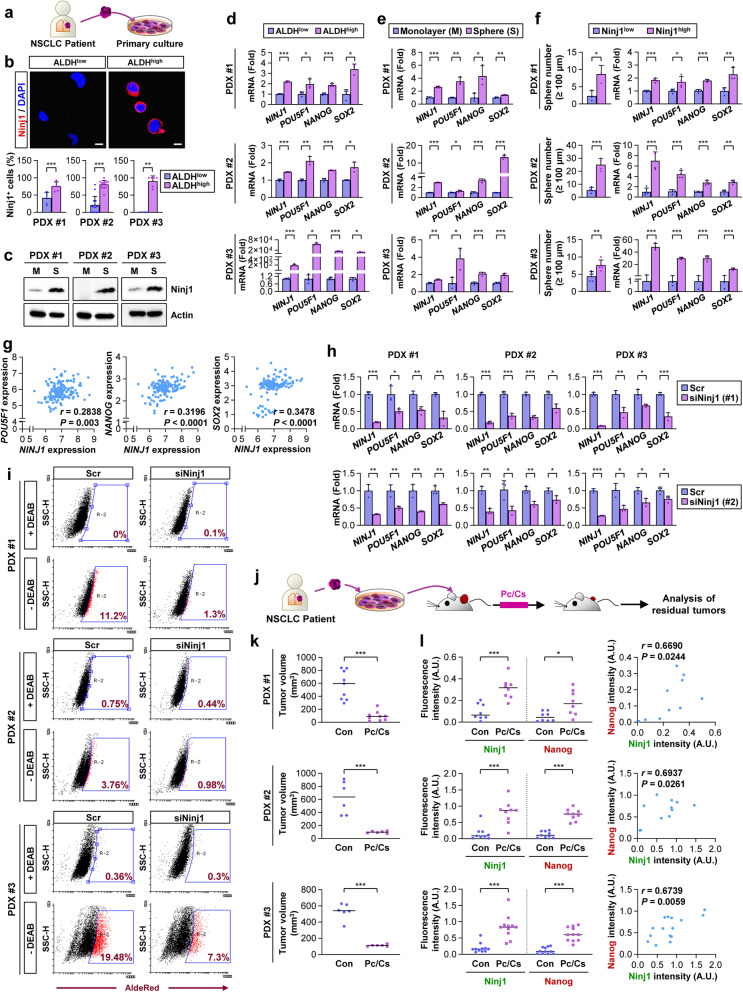
Ninj1 mediates the acquisition of CSC phenotypes in NSCLC cells in patient tumors. **a** Schematic diagram presenting the procedure for isolating primary tumor cells form patient-derived xenograft (PDX) tumors. **b, d** Immunofluorescence (IF) (**b**) and real-time PCR (**d**) analyses of Ninjurin1 (Ninj1) expression in the ALDH^low^ and ALDH^high^ populations from three different PDX tumors. Ninj1^+^ cells were identified using ImageJ software. Scale bars: 20 μm. **c, e** Western blot (**c**) and real-time PCR (**e**) analyses examining the levels of Ninj1 and CSC marker genes, respectively, in primary cultured patient-derived tumor cells grown in monolayer (M) or sphere-forming conditions (S). **f** Sphere formation and real-time PCR analyses assessing sphere formation and the mRNA expression of CSC markers and *NINJ1* in the Ninj1^low^ and Ninj1^high^ population of three PDX-derived primary cultured cancer cells. **g** The Spearman correlation coefficient detailing the relationship between *NINJ1* expression and the expression of stemness markers (*POU5F1*, *NANOG*, and *SOX2*). The correlation was determined by analyzing a GSE77803 dataset. **h, i** Real-time PCR (**h**) and flow cytometric ALDH (**i**) analyses used to determine mRNA expression of *NINJ1* and stemness markers (**h**) and ALDH activity (**i**) in primary cultured patient-derived tumor cells after siRNA-mediated knockdown of Ninj1 expression. **j** Schematic diagram detailing the procedure for analyzing residual PDX tumors after chemotherapy. **k** Changes in the growth of three lung PDX tumors after treatment with a combination of paclitaxel and cisplatin (Pc/Cs; at a dose of 20 mg/kg of paclitaxel and 3 mg/kg of cisplatin, once a week) at the end of the treatment (PDX #1 and PDX #2: 30 days after the start of the treatment; PDX #3: 45 days after the start of the treatment). Con: vehicle-treated control. **l** IF analysis examining the levels of Ninj1 and Nanog expression and their correlation in three PDX tumors treated with Pc/Cs. The Ninj1^+^ cells were identified using ImageJ software. The significance of the correlation was determined by the Spearman rank correlation test. Representative images are presented in Fig. S3g. All in vitro experiments were performed at least three times. The bars represent the mean ± SD; *P < 0.05, **P < 0.01, and ***P < 0.001, as determined by a two-tailed Student’s *t*-test or Mann-Whitney test by comparison to the indicated group. Scr: scrambled siRNA

To assess the clinical relevance of these findings, we analyzed the role of Ninj1 expression in the functional features of CSCs in NSCLC cells obtained from patient-derived tumors (Fig. 4a). IF staining of the ALDH^high^ subpopulation (Fig. 4b) and western blot analysis of the sphere-forming subpopulation (Fig. 4c) within primary cultured patient-derived NSCLC cells revealed elevated Ninj1 protein levels compared to levels in their corresponding controls. Additionally, increased mRNA levels of Ninj1 and CSC marker genes were observed in the ALDH^high^ (Fig. 4d) and sphere-forming (Fig. 4e) subpopulations compared to levels in the controls. Ninj1^high^ subpopulations within the tumors also possessed significantly increased capacities for sphere formation and CSC marker gene expression compared to that of their corresponding Ninj1^low^ subpopulations (Fig. 4f). Analysis of publicly available datasets from patients with NSCLC (GSE77803) further revealed positive correlations between the expression levels of *NINJ1* and CSC markers (Fig. 4g). When Ninj1 expression in primary cultured cells was depleted using siRNAs, the expression of CSC marker genes (Fig. 4h) and ALDH activity (Fig. 4i) were significantly decreased.

Next, we analyzed the role of Ninj1 in the resistance of patient-derived NSCLC cells to various cell death inducers. Similar to the results from NSCLC cell lines, the aforementioned cell death inducers caused marked increases in Ninj1 expression without consistent changes in caspase 1 and caspase 3 cleavage events in the primarily cultured cells (Fig. S3a). Compared to their corresponding Ninj1^low^ subpopulations, Ninj1^high^ subpopulations also possessed similar Ki67 expression but greater anchorage-dependent and anchorage-independent colony formation when cultured in normal culture conditions or in the presence of various cell death inducers (Fig. S3b-f).

We further analyzed mice harboring patient-derived xenograft (PDX) tumors that had been treated with three cycles of a clinically relevant combinatorial chemotherapeutic regimen (i.e., a 7-day regimen comprising paclitaxel and cisplatin for 1 d, followed by a 6-day drug holiday) [33] (Fig. 4j). The Ninj1^high^ populations within the tumors were monitored before and after chemotherapy. The PDX tumors shrank to < 50% of their original volume after treatment (*P* < 0.001) (Fig. 4k). IF staining revealed increased numbers of Ninj1^+^ and Nanog^+^ cells within the residual tumors after chemotherapy compared to those in untreated control tumors, and there was a positive correlation between Ninj1 and Nanog expression (Fig. 4l, S3g). Taken together, these results indicate the presence of distinct Ninj1^high^ subpopulations possessing CSC phenotypes in human NSCLC.

### Ninj1-mediated activation of the canonical Wnt/β-catenin signaling pathway

**Fig. 5:**
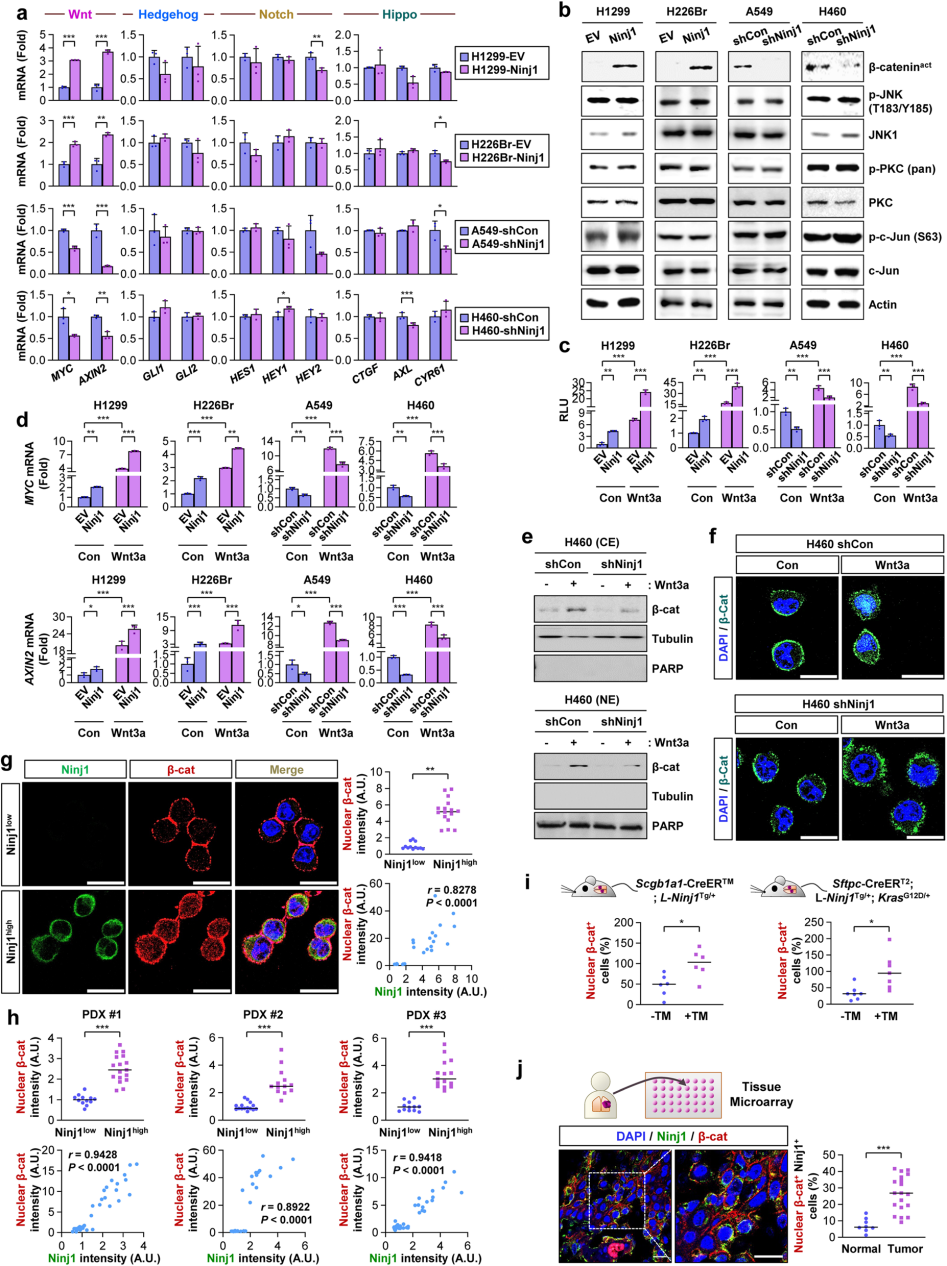
Ninj1 activates the Wnt/β-catenin signaling pathway. **a** Real-time PCR analyses were used to determine changes in the expression of some target genes of the Wnt, Hedgehog, Notch, and Hippo signaling pathways caused by modulation of Ninjurin1 (Ninj1) expression. **b** Western blot analysis examining the expression of the indicated canonical and non-canonical Wnt signaling components in NSCLC cells that achieved upregulation (H1299-Ninj1 and H226Br-Ninj1) or downregulation (A549-shNinj1 and H460-shNinj1) of Ninj1 and in their control cells. β-catenin^act^: active β-catenin. **c, d** The TOPFlash luciferase reporter assay (**c**) and real-time PCR analysis examining *MYC* and *AXIN2* (**d**) in the indicated stable NSCLC cells with overexpression or knockdown of Ninj1 expression in the absence or presence of Wnt3a conditioned medium (Wnt3a). **e** Western blot analysis examining the basal and Wnt3a-induced nuclear translocation of active β-catenin (β-catenin^act^) in H460-shCon and H460-shNinj1 cells. **f** Immunofluorescence (IF) analysis examining the basal and Wnt3a-induced nuclear translocation of β-catenin (β-cat) in H460-shCon and H460-shNinj1 cells. **g** IF analysis assessing the levels of Ninj1 and β-catenin (β-cat) expression and their correlation between Ninj1 and nuclear β-catenin in the Ninj1^high^ and Ninj1^low^ subpopulations in H460 cells. The significance of the correlation was determined using the Spearman rank correlation test. **h** IF analysis examining the level of Ninj1 and β-catenin expression in Ninj1^high^ and Ninj1^low^ subpopulations of primary cultured patient-derived tumor cells. The significance of the correlation was determined using the Pearson correlation test (PDX #1 and #3) and the Spearman rank correlation test (PDX #2). Representative IF images are presented in Fig. S5b. **i** IF analysis indicating the levels of Ninj1 and nuclear β-catenin (β-cat) expression in CCSP^+^ club cells and SPC^+^ type II alveolar epithelial cells (AT2s) and their correlation. The Ninj1^+^ cells were identified using ImageJ software. The significance of the correlation was determined using the Spearman rank correlation test. Representative IF images are presented in Fig. S5c. **j** IF analysis examining the level of Ninj1 and nuclear β-catenin (β-cat) expression in a tissue microarray of patient-derived normal and NSCLC tissues. All in vitro experiments were performed at least three times. The bars represent the mean ± SD; *P < 0.05, **P < 0.01, and ***P < 0.001, as determined by a two-tailed Student’s t-test or Mann-Whitney test by comparison to the indicated group or one-way ANOVA with Tukey’s post-hoc test (**c, d**). Scale bars: 50 μm (**f, g, j**). Con: control conditioned medium (**c, d, f**). TM: tamoxifen

To elucidate the mechanism by which Ninj1 mediates the acquisition of CSC phenotypes, we investigated the effects of Ninj1 expression on the Wnt/β-catenin, Notch, and Hedgehog pathways that play critical roles in stem cell function [12, 13]. The expression of the representative target genes of the Wnt/β-catenin signaling pathway was significantly upregulated in H1299-Ninj1 and H226Br-Ninj1 cells and was decreased in H460-shNinj1 and A549-shNinj1 cells compared to levels in their respective control cells, while the expression levels of the Notch, Hedgehog, and Hippo pathways did not exhibit consistent changes in these cells (Fig. 5a). Notably, Ninj1 induced a decrease in the level of active β-catenin (β-catenin^act^) that functions to mediate canonical Wnt signaling [9], while no detectable changes were observed in non-canonical Wnt signaling mediators, including phosphorylated forms of c-Jun N-terminal kinase (JNK), protein kinase C (PKC), and c-Jun, thus indicating Ninj1-mediated regulation of the canonical Wnt signaling pathway (Fig. 5b). We then explored the direct evidence supporting the functional involvement of Ninj1 in the activation of the Wnt/β-catenin signaling pathway. The TOPFlash luciferase reporter assay (Fig. 5c, left), a common tool used to measure the activation of the Wnt/β-catenin signaling pathway [50], and real-time PCR analysis of *AXIN2* and *MYC* expression (Fig. 5d, left) as representative target genes of the pathway [51] revealed increased activation of the Wnt/β-catenin pathway in H1299-Ninj1 and H226Br-Ninj1 cells compared to that in their corresponding control cells. Ninj1-mediated Wnt/β-catenin signaling is further enhanced by the addition of exogenous Wnt3a, a Wnt ligand that activates the canonical Wnt signaling pathway and promotes lung cancer progression [16]. In contrast, A549-shNinj1 and H460-shNinj1 cells exhibited significant attenuation in Wnt/β-catenin signaling events compared to that of their corresponding control cells (Fig. 5b-d, right; Fig. S4a). Additionally, western blot (Fig. 5e) and IF (Fig. 5f) analyses revealed that the Wnt3a-mediated nuclear localization of β-catenin (Fig. 5e) and β-catenin (Fig. 5f) was markedly suppressed in H460-shNinj1 cells compared to that in their corresponding control cells. We further observed significantly greater levels of nuclear β-catenin expression in the FACS-sorted Ninj1^high^ populations from H460 cells (*P* < 0.001) (Fig. 5g) and three different PDXs (*P* < 0.001) (Fig. 5h, S4b) compared to that observed in their corresponding Ninj1^low^ populations. CCSP^+^ club cells in lung tumors from urethane-exposed *Scgb1a1*-CreER^TM^;L-*Ninj1*^Tg/+^ mice and SPC^+^AT2s in lung tumors from *Sftpc*-CreER^T2^;L-*Ninj1*^Tg/+^;*Kras*^G12D/+^ mice also possessed significantly higher nuclear β-catenin expression compared to that in the corresponding control cells from urethane-exposed *Scgb1a1*-CreER^TM^;LSL-*Ninj1*^Tg/+^ mice (*P* = 0.0138) and *Sftpc*-CreER^T2^;LSL-*Ninj1*^Tg/+^;*Kras*^G12D/+^ mice (*P* = 0.0107), respectively (Fig. 5i, S4c). Using IF analyses of an NSCLC tissue microarray (*n* = 40), we further demonstrated significant increases in the nuclear β-catenin^+^Ninj1^+^ populations in tumors compared to those in normal tissues (*P* < 0.001) (Fig. 5j). These results suggested that Ninj1 is involved in the activation of the canonical Wnt/β-catenin signaling pathway in NSCLC.

Given the general role of the Wnt/β-catenin signaling pathway in human cancers [9, 10, 14], we assessed the pathological role of Ninj1 in histologically distinct epithelial tumors, including breast and colon cancers. Public data analysis revealed that *NINJ1* expression was a poor prognostic factor in these cancers (Fig. S5a). Positive correlations between the expression levels of *NINJ1* and *SOX2* were observed in these cancers (Fig. S5b). We further validated the significantly increased CSC marker gene expression in the Ninj1^high^ population from patients with breast and colorectal cancers compared to that in the Ninj1^low^ population from the corresponding tumors (Fig. S5c). Hence, Ninj1 may be implicated in the development and progression of various human cancers.

### Ninj1-mediated promotion of the assembly of the LRP6-FZD2 signalosome

**Fig. 6:**
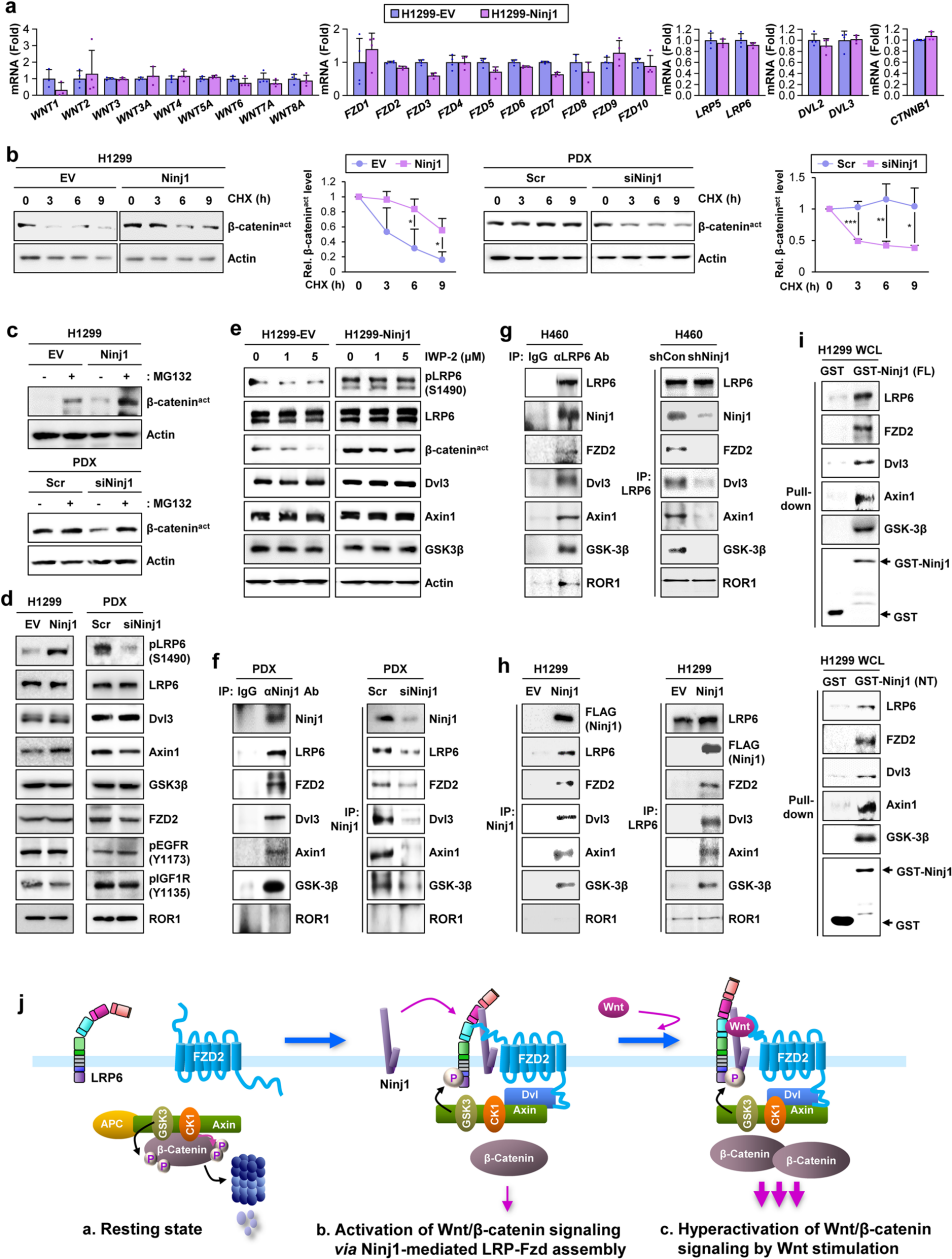
Ninj1 is a key modulator of the activation of Wnt/β-catenin pathway in NSCLC cells. **a** Real-time PCR analyses examining mRNA expression of Wnt ligands (*WNT1-WNT8A*), Frizzled receptors (*FZD1-FZD10*), LRP (*LRP5* and *LRP6*), DVL (*DVL2* and *DVL3*), and β-catenin (*CTNNB1*) in H1299-EV and H1299-Ninj1 cells. **b**, **c** Western blot analysis examining active β-catenin (β-catenin^act^) protein in the indicated NSCLC cells after treatment with cycloheximide (CHX, 100 μg/mL) for the indicated time-points (**b**) or in cells treated with MG132 (10 μM) (**c**). **d** Western blot analysis examining the expression of the indicated canonical Wnt/β-catenin signaling components and the phosphorylation of EGFR, IGF-1R, and ROR1 in H1299-Ninj1, PDX-siNinj1, and their control cells. **e** Western blot analysis examining the expression of the indicated canonical Wnt/β-catenin signaling components in H1299-EV and H1299-Ninj1 cells in response to treatment with IWP-2. **f-h** Western blot analysis of IgG, anti-Ninj1 (**f, h**), and anti-LRP6 (**g, h**) immunoprecipitates (IPs) for the indicated Wnt/β-catenin signaling proteins to determine interactions among Ninj1, LRP6, FZD2, and other Wnt/β-catenin signaling components in the indicated NSCLC cells. **i** Pull-down assays assessing the interaction between glutathione-agarose-bound full-length (FL) or N-terminal (NT) domain of GST-Ninj1 and the Wnt signaling components, including LRP6, FZD2, Dvl3, Axin1, and GSK-3β, in H1299 whole cell lysates (WCL). **j** Schematic model of the Wnt/β-catenin signaling pathway in the absence or presence of Ninj1. In the absence of Ninj1 (a resting state), Wnt signaling is inactivated by the destruction complex-mediated β-catenin destabilization. Ninj1 promotes the assembly of the LRP6 signalosome, thus leading to a weak activation of the Wnt/β-catenin signaling pathway. Wnt ligand fully activates the Wnt/β-catenin signaling pathway in the presence of Ninj1. All in vitro experiments were performed at least three times. The bars represent the mean ± SD; *P < 0.05, **P < 0.01, and ***P < 0.001, as determined by a two-tailed Student’s t-test

We investigated the mechanism by which Ninj1 activates the Wnt/β-catenin signaling pathway. The mRNA levels of Wnt1-8a, seven-span FZD1–10, single-span LRP5 and LRP6, cytosolic effectors Dvl2 and Dvl3, and β-catenin were either decreased or remained unchanged in H1299-Ninj1 cells compared to those in H1299-EV cells (Fig. 6a, S6a), thus suggesting that Ninj1-mediated Wnt/β-catenin signaling occurs through post-transcriptional mechanisms. As Wnt binding to two cell surface receptors (LRP5/6 and FZD) is known to induce LRP5/6 phosphorylation through the intermediation of Disheveled (Dsh; Dvl in mammals), Axin, and its associated kinase GSK-3 [9, 10] to thus release β-catenin from the multiprotein destruction complex [52], we hypothesized that Ninj1 may regulate β-catenin stability. Indeed, upon treatment with the protein synthesis inhibitor cycloheximide [53], the half-life of β-catenin was significantly increased in H1299-Ninj1 cells and was decreased in H460-shNinj1 cells and PDX-derived primary tumor cells transfected with Ninj1 siRNAs (PDX-siNinj1) compared to that in the corresponding control cells (Fig. 6b, S6b, S6c). Moreover, pretreatment with MG132 increased β-catenin levels in H1299-EV, H460-shNinj1, and PDX-siNinj1 cells compared to levels in H1299-Ninj1, H460-shCon, and PDX-Scr cells, respectively (Fig. 6c, S6d). Notably, compared to control cells, H1299-Ninj1, H460-shNinj1, and PDX-siNinj1 cells exhibited increased and decreased LRP6 phosphorylation, respectively, without detectable changes in LRP6, FZD2, Dvl3, Axin1, and GSK-3β protein expression (Fig. 6d, S6e). Moreover, Wnt3-mediated LRP6 phosphorylation was markedly enhanced in H1299-Ninj1 cells and was attenuated in H460-shNinj1 cells (Fig. S6f). In contrast, the phosphorylation of other receptor tyrosine kinases, including epidermal growth factor receptor (EGFR) and insulin-like growth factor receptor (IGF-1R), was not affected by the modulation of Ninj1 expression (Fig. 6d, S6e). We then assessed if Ninj1 can induce ligand-independent activation of the LRP6/β-catenin signaling cascade by utilizing IWP-2, a Wnt/β-catenin inhibitor that blocks Porcn-mediated Wnt palmitoylation [54]. IWP-2 treatment effectively blocked LRP6 phosphorylation and β-catenin activation in H1299-EV cells (Fig. 6e). In contrast, Ninj1-mediated LRP6 phosphorylation and β-catenin activation remained unchanged in H1299-Ninj1 cells following IWP-2 treatment. Thus, Ninj1 appears to possess the capacity to activate LRP6 in a ligand-independent manner.

We then determined if Ninj1 interacts with LRP signaling components. Co-immunoprecipitation analyses revealed an association between Ninj1 and LRP6, FZD2, Dvl3, Axin1, and GSK-3β in PDX-Scr and H460 cells, and this association did not occur in PDX-siNinj1 and H460-shNinj1 cells (Fig. 6f, S6g). LRP6 co-immunoprecipitation with Ninj1, FZD2, Dvl3, Axin1, and GSK-3β was also observed in H460 cells but not in H460-shNinj1 cells (Fig. 6g). Co-immunoprecipitation of Ninj1, LRP6, FZD2, Dvl3, Axin1, and GSK-3β was also observed in H1299-Ninj1 cells and not in H1299-EV cells (Fig. 6h). ROR1, another Wnt receptor [9], was observed to interact with LRP6 [55]. Indeed, LRP6 and not Ninj1 co-immunoprecipitated with ROR1 (Fig. 6f-h). A pull-down assay using GST-tagged Ninj1 recombinant proteins revealed that Ninj1 as the full-length (FL) protein or N-terminal (NT) domain was associated with LRP6, FZD2, Dvl3, Axin1, and GSK-3β (Fig. 6i). These findings suggest that the Ninj1-mediated Wnt/β-catenin signaling pathway occurs through the assembly of the LRP6-FZD2 signalosome.

## Discussion

Despite the advent of antineoplastic drugs, NSCLC remains the leading cause of cancer-related deaths [1]. Based on the proposed roles of CSCs in tumor development, progression, and drug resistance [5, 13], determining the molecular functions of CSCs, understanding the mechanisms underlying their biology, and developing CSC-targeting therapeutic strategies will provide logical approaches for the treatment of various human cancers, including NSCLC [13]. Herein, we identified an NSCLC CSC population in which Ninj1 activates the canonical Wnt/β-catenin signaling pathway to ensure survival under conditions of microenvironmental insults. The N-terminal domain of Ninj1 associates with LRP6 and FZD2 receptors that exist in inactive forms on the resting cell surface (Fig. 6j-a) to facilitate the local recruitment of Dvl, Axin, and GSK-3β, the phosphorylation of LRP6, and the subsequent nuclear translocation of β-catenin, ultimately leading to the transcriptional upregulation of genes involved in resistance to hazardous microenvironments (Fig. 6j-b). Exogenous Wnt signaling enhanced the scale of this signaling (Fig. 6j-c). This observation suggests that the Ninj1-LRP6-FZD2 assembly operates via the Wnt/β-catenin signaling pathway to facilitate the survival of NSCLC CSCs in hostile environments.

CSCs may appear after oncogenic transformation of normal stem cells or early stem cell progenitors or after dedifferentiation of genetically or epigenetically altered differentiated cells [4, 6, 56]. Several molecules such as CD24, CD44, and CD133 have been recognized as CSC markers for certain cancer types [13]; however, the roles of these markers are ambiguous. For example, pancreatic cancer cells possessing high CD24 expression were determined to induce tumor initiation [57], while low levels of CD24 expression were observed in breast CSCs [58]. Moreover, these markers are not reliably expressed in different types of cancer, including NSCLC [59]. Hence, we aimed to identify molecules that confer functional features of CSCs to NSCLC cells. Our data analysis of human and mouse lung tissues and the results published within publicly available datasets suggest that Ninj1 is a regulator of lung tumor development and progression and provides a marker for poor prognosis in patients with NSCLC. In support of this notion, our study used two mouse models where lung tumor development was initiated by oncogenic *KRAS* mutation or TC exposure, and we observed that Ninj1 overexpression in putative lung tumor-initiating cells, including SPC^+^ AT2s and CCSP^+^ club cells, promoted lung tumor growth that resulted in severe morbidity and mortality.

We then investigated how Ninj1 functions as a driver of lung tumorigenesis. A recent report suggested a role for Ninj1 in inducing plasma membrane rupture in macrophages in response to inducers of pyroptotic, necrotic, and apoptotic cell death such as depletion of nutrients, hypoxia, or exposure to chemotherapeutic drugs [22]. However, in the current study, Ninj1^high^ subpopulations from NSCLC cell lines and PDX tumors exhibited significantly greater survival capacity against programmed cell death inducers [45] without a detectable change in proliferation rate. Therefore, responses to Ninj1 expression appear to be highly cell type-dependent, and Ninj1^high^ NSCLC cells may represent a distinct subpopulation harboring a prominent survival potential under environmental insults. Our subsequent findings using Ninj1^high^ and Ninj1^low^ subpopulations from NSCLC cell lines and PDX tumors revealed a positive correlation between Ninj1 expression and the functional features of CSCs (i.e., high ALDH activity, tumorsphere formation under particular culture conditions, and expression of SOX2, Nanog, and Oct4) [60]. Moreover, forced overexpression of Ninj1 appeared to endow NSCLC cells with the functional features of CSCs and also with survival capacity in in vitro and in vivo microenvironments featuring cell death inducers. In contrast, loss-of-Ninj1 expression attenuated the survival capacities of these cells in hazardous environments. Hence, although the implication of other mechanisms has not been excluded, Ninj1 appears to promote lung tumorigenesis by conferring survival capacities to NSCLC CSCs under environmental insults.

We next sought to determine how Ninj1 confers NSCLC CSCs with prominent survival capacities. We identified a previously undiscovered mechanism by which Ninj1 stimulates the canonical Wnt/β-catenin signal transduction pathway in the absence of ligands. The single-pass Wnt co-receptor LRP6 has been proposed to possess a multi-modular ectodomain that allows for the formation of a huge multi-molecular assembly known as the “LRP6 signalosome” upon sensing of the Wnt signal [61–63]. Our results indicate that the N-terminal extracellular domain of Ninj1 forms a complex with LRP6 and FZD2. Wnt-independent activation of canonical Wnt/β-catenin signaling has been well documented in cells where forced overexpression of mutant *LRP6* lacks the entire ectodomain [64]. Spontaneous Wnt/β-catenin signaling was also observed in response to WT *LRP6*, and the degree of activation was inversely associated with ectodomain length [65]. It has been proposed that four tandem β-propeller–EGF-like domain (PE) modules of the LRP6 ectodomains occupy substantial space on the cell surface, thus prohibiting signaling in its resting state, and engagement of Wnt ligands or antagonists induces conformational changes in the LRP6 ectodomain that facilitate the regulation of Wnt/β-catenin signaling activation [66]. Hence, it is reasonable to speculate that the Ninj1 N-terminal domain possesses the intrinsic capacity to function as a signaling platform by inducing sequential events, including conformational changes in the ectodomain of LRP6 and spontaneous assembly of the LRP6 signalosome, to thus induce β-catenin-mediated transcriptional upregulation of Wnt target genes. Wnt/β-catenin signaling prevents p53-mediated apoptosis, inhibits mitochondrial release of cytochrome c, and elevates the expression of anti-apoptotic proteins [67]. Nuclear β-catenin was demonstrated to induce the transcription of genes involved in immune evasion such as *CCL4*, *CD47*, and *CD274* [68]. Therefore, the Ninj1-mediated survival of NSCLC CSCs in hostile environments may, at least in part, be due to the pro-survival function of the Wnt/β-catenin signaling pathway.

The insights gained from the results of our current research study convey significant translational connotations. Ninj1 is a potential surface biomarker for CSCs, and Ninj1-targeted therapeutic interventions may be effective for eradicating CSCs. Additionally, Ninj1 can serve as a predictive biomarker for therapeutic interventions targeting the Wnt/β-catenin signaling pathway. Given that Ninj1 is overexpressed in a range of histologically distinct epithelial tumors, including lung, breast, and colon cancers, and that the role of the Wnt/β-catenin signaling pathway in human cancers in general [9, 10, 14], the pathophysiological role of Ninj1 has been implicated in various human cancers. Therefore, Ninj1-targeting therapeutics can be combined with other anticancer drugs to treat diverse human cancers.

## Conclusions

We demonstrated that Ninj1 functions as a critical regulator of Wnt/β-catenin signaling to confer NSCLC CSCs with survival potential in hazardous environments. Our findings provide a deeper and broader understanding of the biology of NSCLC CSCs and suggest potential novel therapeutic strategies for the treatment of NSCLC. Considering the intra- and inter-patient variability of tumors, future studies utilizing clinical tissues are required to clearly elucidate the impact of Ninj1 as a marker for the identification and characterization of CSCs and the role of Ninj1 crosstalk with Wnt/β-catenin signaling in CSC-mediated lung tumor development. Further structural studies are warranted to answer other important questions, including the specificity and stoichiometry of Ninj1 with the LRP6 and FZD2 complex under Wnt-off and Wnt-on conditions and also the upstream effectors that control the dynamic disassembly/assembly of the Ninj1-LRP6-FZD2 complex.

## Supplementary Information

**Additional file 1.**

## Data Availability

All data generated or analyzed during this study are included in this published article and its supplementary information files.

## Abbreviations

ALDH: Aldehyde dehydrogenase
APC: Adenomatous polyposis coli
AT2: Alveolar type II epithelial cells
CKI: Casein kinase I
Col: Collagen
CSC: Cancer stem-like cells
EGFR: Epidermal growth factor receptor
ER: Estrogen receptor
EV: Empty vector
FACS: Fluorescence-activated cell sorting
FL: Full-length
Fn: Fibronectin
FZD: Frizzled
GSK-3: Glycogen synthase kinase-3
GST: Glutathione S-transferase
IF: Immunofluorescence
IGF-1R: Insulin-like growth factor 1 receptor
IHC: Immunohistochemistry
JNK: c-Jun N-terminal kinase
LRP: Lipoprotein receptor-related protein
LSL: LoxP-Stop-LoxP
Ninj1: Ninjurin1
NSCLC: Non-small cell lung cancer
NHBE: Normal human bronchial epithelium
NT: N-terminal
PDX: Patient-derived xenograft
PE: β-propeller-EGF-like domain
PKC: Protein kinase C
ROR1: Receptor tyrosine kinase like orphan receptor 1
SC: Stem cell
SPC: Surfactant protein C
TC: Tobacco carcinogen
Tg: Transgenic
TM: Tamoxifen
WT: Wild type
