# Supplementary material for: Ninjurin1 drives lung tumor formation and progression by potentiating Wnt/β-Catenin signaling through Frizzled2-LRP6 assembly
Source: J Exp Clin Cancer Res. 2022 Apr 8;41:133. doi: 10.1186/s13046-022-02323-3 (PMC8991582; doi:10.1186/s13046-022-02323-3)
Supplement: Supplementary file 1 — Additional file 1. [file 13046_2022_2323_MOESM1_ESM.pdf]

Supplementary Materials

Ninjurin1 drives lung tumor formation and progression by potentiating Wnt/β-Catenin signaling through Frizzled2-LRP6 assembly

Seung Yeob Hyun, Hye-Young Min, Ho Jin Lee, Jaebeom Cho, Hye-Jin Boo, Myungkyung Noh, Hyun-Ji Jang, Hyo-Jong Lee, Choon-Sik Park, Jong-Sook Park, Young Kee Shin, Ho-Young Lee

Methods

Preparation of control and Wnt3a conditioned media

Control and Wnt3a conditioned media were obtained by using L and L-Wnt3a cells, respectively, according to the protocol available in the Wnt homepage (http://web.stanford.edu /group/nusselab/cgi-bin/wnt/).

In silico analysis

We used publicly available datasets deposited in the GEO (National Center for Biotechnology Information): GSE31210 for evaluation of the level of the *NINJ1* expression in NSCLC tumors derived from NSCLC patients by comparison with that in the corresponding control tissues; GSE31210, GSE9893, and GSE17537 for analysis of the association of the *NINJ1* expression with prognosis of patients with lung, colon, and breast cancer; GSE77803, GSE9893, and GSE85043 for analysis of the correlation of the *NINJ1* expression with CSC markers (*POU5F1*, *NANOG*, or *SOX2*). Raw data comprising gene expression levels and clinical information for each patient sample (such as histology, survival status, and duration of survival) were manually downloaded and analyzed. A Kaplan-Meier survival curve was used to show differences in the survival of lung cancer patients. For survival analysis, the top and bottom 25% percentile were used to determine the $Ninj1^{high}$ and $Ninj1^{low}$ groups, respectively. The log-rank test was used to determine significance.

Immunohistochemistry

Sections of formalin-fixed and paraffin-embedded tissue specimens were deparaffinized, rehydrated, and then subjected to antigen retrieval using the citrate-based antigen unmasking solution (Vector Laboratories). After treatment with 0.3% hydrogen peroxide solution, slides were incubated with blocking buffer (5% normal serum in TBS containing 0.025% Triton X-100) for 1 h at room temperature. Slides were incubated with primary antibodies overnight at 4ºC and then

with a biotinylated secondary antibody (Bethyl laboratories) for 1 h at room temperature. Solutions A and B (ABC-Elite, Vector Laboratories) were added simultaneously for 30 min, and signals were detected using a 3,3'-diaminobenzidine (DAB) substrate kit (Vector Laboratories). Slides were further counterstained with hematoxylin.

Immunofluorescence

Sections of formalin-fixed, paraffin-embedded (FFPE) tissue specimens were deparaffinized, rehydrated, and treated with citrate-based antigen unmasking solution (Vector Laboratories, Burlingame, CA, USA) for antigen retrieval. The slides were treated with 0.3% hydrogen peroxide solution and then incubated with blocking solution (5% normal serum in TBS containing 0.025% Triton X-100) for 1 h at room temperature. The slides were incubated with primary antibodies (1:100 dilution) overnight at 4ºC. The slides were washed multiple times with wash buffer (TBS containing 0.025% Triton X-100), incubated with fluorochrome-labeled secondary antibodies (Thermo Fisher Scientific) for 1 h at room temperature, and then washed several times with wash buffer. The slides were counterstained with 4′,6-diamidino-2-phenylindole (DAPI) and observed under a fluorescence microscope (Zeiss Axio Observer Z1, Carl Zeiss AG, Oberkochen, Germany).

MTT assay

Cells were seeded into 96-well plates at a density of 2×10^3 to 1×10^4 cells/well and allowed to attach for 24 h. Cells were treated with vehicle or the indicated concentrations of test compounds diluted in complete media for 2 days, after which they were treated with MTT solution (final concentration of 500 μg/mL) and incubated for 2-4 h at 37°C. The formazan products were dissolved in DMSO, and the absorbance of each well was measured at 570 nm. The data are presented as a percentage of the control group.

Sphere formation assay

Cells were seeded on ultra-low attachment 96-well plates (Corning, Corning, NY, USA) in spheroid medium [DMEM-F12, supplemented with B27 supplements (Thermo Fisher Scientific, Waltham, MA, USA), EGF, bFGF, and antibiotics]. Cells were incubated at 37°C and 5% CO_2 for 2 weeks or until spheres formed and reached above 150 μm^2. Spheres were imaged, and the diameter of spheres and the number of spheres above 30 or 100 μm in diameter were determined using ImageJ software (National Institutes of Health, Bethesda, MA, USA).

Aldehyde dehydrogenase (ALDH) assay

1 x 10^6 NSCLC cells were suspended in AldeRed buffer and stained with AldeRed A588 at 37°C for 40 min. Each group contained a blank sample (AldeRed A588 alone) and a positive control sample (AldeRed A588 plus DEAB). The fluorescence intensity was obtained by flow cytometric analysis, and the sorting gates were established using a sample with DEAB treatment (negative control). The $ALDH^{high}$ and $ALDH^{low}$ populations were sorted using a FACS Aria III flow cytometer (BD Biosciences) for further in vitro experiments.

Anchorage-dependent colony formation assay

Cells were seeded into 6-well plates at a density of 300 cells/well and treated for two weeks with various concentrations of test materials diluted in complete medium. The drug-containing medium was changed once or twice a week. After incubation, colonies were fixed with 100% methanol, stained with 0.02% crystal violet solution, and washed with deionized water several times. Colonies were imaged and counted using ImageJ software.

Soft agar colony formation assay

Cells were mixed with sterile 1% agar solution (final concentration of 0.4%) and poured onto 1% base agar in 24-well plates. Test materials diluted in complete medium was added to the agar after solidification of the top agar. Cells embedded in the top agar were incubated for 2 weeks at 37°C

with 5% CO_2, and the medium was changed twice a week. After incubation, colonies were stained with the MTT solution (final 250-500 µg/mL). Colonies were imaged and counted using ImageJ software.

Western blot analysis

Total cell lysates were prepared with modified RIPA lysis buffer (50 mM Tris-HCl [pH 7.4], 150 mM NaCl, 1 mM EDTA, 0.25% sodium deoxycholate, 1% Triton X-100) containing various protease and phosphatase inhibitors (100 mM NaF, 5 mM Na_3VO_4, 1 mM PMSF, 1 µg/mL aprotinin, 1 µg/mL leupeptin, and 1 µg/mL pepstatin). Equal amounts of protein (25-50 µg) were subjected to SDS-PAGE though 6-12% gels and electrophoretically transferred onto polyvinylidene difluoride (PVDF) membranes (ATTO Corp., Tokyo, Japan). Membranes were submerged into blocking buffer [5% nonfat dry milk in Tris-buffered saline (TBS) containing 0.01% Tween-20 (TBST)] for 1 h at room temperature before they were incubated overnight at 4°C with primary antibodies diluted in 3% BSA in TBST (1: 1,000). Membranes were then washed multiple times with TBST and incubated with secondary antibodies diluted in 5% nonfat dry milk in TBST (1: 5,000) for 1 h at room temperature. Finally, the membranes were washed multiple times with TBST, and protein bands were visualized using an enhanced chemiluminescence (ECL) detection kit (Thermo Fisher Scientific).

Real-time PCR

Total RNA was prepared using an easy-BLUE total RNA extraction kit (Intron Biotechnology, Sungnam-si, Kyunggi-do, Republic of Korea) according to the manufacturer's recommended procedure. We used a SYBR Green-based qPCR master mix solution (Enzynomics, Daejeon, Republic of Korea) and gene-specific primers. All real-time PCR assays were performed on an Applied Biosystems 7300 Real-Time PCR System (Thermo Fisher Scientific). The following thermocycler conditions for real-time PCR were applied: pre-incubation at 95°C for 15 min; 40-70

cycles of 95°C for 10 sec, 60°C for 15 sec, and 72°C for 30 sec; and a final melt curve analysis to determine reaction specificity. Relative quantification of mRNA expression was performed using the comparative CT (cycle threshold) method as described in a previous report[1]. The primer sequences used in the PCR assays are shown in **Table S3**.

Plasmids, shRNAs, and Transfection

The plasmid constructs for hNinj1 expression are described in our previous report [2]. The plasmid construct for the FLAG-tagged hNinj1 protein was generated by using pCS+GFP hNinj1 vector as a template and subcloning them into the pCMV-Tag2B vector using the following primers: FLAG Ninj1 forward: 5'-TGTGAATTCATGGACTCGGGAACC-3', reverse: 5'-TATCTCGAGCTACTGCTGGGGTGC-3'.

The plasmid constructs for the myc-tagged full-length hNinj1 protein and its domain constructs were generated by cloning them into the pCS2+MT vector. NT (1-100 aa), ∆Cyto (1-100 linked with 111-152 aa) and CT (111-152 aa) domain constructs were generated using the template pCS2+MT-FL-hNinj1 and the following primers: NT forward: 5'-CAGGAATTCGATGGACTCGGGAAC-3', reverse: 5'-TCTCGAGTCAGAAGATGAGCAGCAC-3'; CT forward: 5'-CAGGAATTCGAAGCACGCCAAGCTG-3', reverse: 5'-TATCTCGAGCTACTGCTGGGGTGC-3'. For ∆Cyto mutant hNinj1, PCR products of NT fragments and fragments were ligated and subcloned into pCS2+MT vector.

For GST-tagged bacterial protein expression, pCMV-Tag2B-hNinj1 was subcloned into the pGEX-4T-2. NT Ninj1 mutant was generated using the template pGEX-4T-2-FL-hNinj1 with the following primers: NT GST Ninj1 forward: 5'-CAGGAATTCCAATGGACTCGGGAAC-3', reverse: 5'-TCTCGAGTCAGAAGATGAGCAGCAC-3'.

His-tagged bacterial protein expression, LRP6 and FZD2 domain constructs were subcloned into the pET32a. LRP6 constructs were generated by using LRP6-pCS2 vector as a template and FZD2 construct was generated by using pCMV6-AC-GFP FZD2. PE1 LRP6 (1-328

aa), PE2 LRP6 (329-631 aa), PE3 LRP6 (632-932 aa), PE4 LRP6 (933-1247 aa) and Ext FZD2 (24-247 aa, no signal peptide) were generated using the following primers: PE1 LRP6 forward: 5'-TATGGATCCATGGGGGCCGTCCTG-3', reverse: 5'-TATGTCGACTCATGTGGCACCATC-3'; PE2 LRP6 forward: 5'-TATGGATCCGAATTATTGCTTTTA-3', reverse: 5'-TATGTCGACTCACTCTGGGACAAT-3'; PE3 LRP6 forward: 5'-TATGGATCCGCTTTCCTTTTGTTT-3', reverse: 5'-TATGTCGACTCAAGGAGCACTACA-3'; PE4 LRP6 forward: 5'-TATGGATCCACGACTTTCCTGCTC-3', reverse: 5'-TATGTCGACTCATGGAGGTTCTCC-3'; Ext FZD2 forward: 5'-ATGAATTCCAGTTCCACGG-3', reverse: 5'-ATAAAGCTTTTACCAGAGGCGC-3'.

The pCS+GFP hNinj1 expression vector was kindly provided by Dr Kyu-Won Kim (Seoul National University, Seoul, Korea). The LRP6-pCS2 vector was purchased from Addgene (Plasmid #27242, Watertown, MA, USA). The pCMV6-AC-GFP FZD2 vector was purchased from OriGene Technologies (Cat #: RG220921, Rockville, MD, USA) (Origene-Technologies). The scrambled small interfering RNA (siRNA) for silencing hNinj1 and control siRNA were purchased from Integrated DNA Technologies (Coralville, IA, USA).

For transient transfection, cells were transfected with expression vectors or siRNAs using the JetPrime transfection reagent (Polyplus-Transfection SA). To generate stable cell lines knocked down Ninj1 expression, H460 and A549 cells were transduced with lentiviral particles containing control vector (shCon; pLKO.1) or *NINJ1* shRNAs (Sigma-Aldrich). The stable transfectants were selected by culturing cells in a medium containing 1 – 2 μg/mL puromycin for 3 weeks. To establish stable cell lines overexpressing Ninj1, H1299 and H226Br cells were transfected with pCMV-Tag2B (EV) or pCMV-Tag2B-Ninj1 (Ninj1) vectors using JetPRIME for 48 h. Transfected cells were selected using G418.

Immunoprecipitation and pulldown assay

For immunoprecipitation analysis, cells were washed with ice-cold PBS twice and then harvested

by IP lysis buffer [20 mM Tris-HCl (pH 7.5), 150 mM NaCl, 0.5% NP-40, 1 mM $MgCl_2$, 10% glycerol, 100 mM NaF, 5 mM Na_3VO_4, 1 μg/mL aprotinin, 1 μg/mL leupeptin, and 1 μg/mL pepstatin] for 10 min on ice. After centrifugation at 13,000 rpm for 10 min at 4°C, supernatants were harvested, and protein concentration was determined by the BCA assay. 1 mg of protein was immunoprecipitated with primary antibodies overnight at 4°C in lysis buffer. Protein G agarose beads were added and incubated for additional 2 h. The beads were collected by centrifugation (3,000 rpm for 2 min at 4°C) and washed six times (three times with lysis buffer and three times with PBS). Bound proteins were extracted by boiling with 5x SDS-PAGE sample buffer for 5 min at 95°C. Proteins were resolved by SDS-PAGE, transferred onto PVDF membranes, and then subjected to Western blot analysis as described above.

The expression and purification of hexahistidine (6xHis, His)-tagged recombinant LRP6 and FZD2 or glutathione-S-transferase (GST)-tagged Ninj1 proteins were performed as described previously [3]. For a pull-down assay, the recombinant Ninj1 protein bound to the Ni-NTA agarose or the recombinant vimentin protein bound to the glutathione-agarose were incubated with 1 mg of cell lysates or purified proteins in TNE binding buffer [50 mM Tris-HCl (pH 8.0), 120 mM NaCl, and 0.1 M EDTA) with rotation for 2 h or overnight at 4°C. The pull-down complexes were centrifuged and washed thrice with the lysis buffer before being resolved using SDS-PAGE and analyzed by Western blot analysis.

Limiting dilution assay

Cells were harvested by trypsinization. Live cells, as confirmed using a trypan blue exclusion assay, were diluted in PBS, mixed with Matrigel (ratio 1:1), and then inoculated into the right flanks of NOD/SCID mice. The incidence of tumor formation was determined. Tumor-initiation fraction of vehicle- or Evo-treated groups was determined by using Extreme Limiting Dilution Analysis (ELDA) online software (http://bioinf.wehi.edu.au/software/elda/)[4].

Hanging drop assay

Cells (suspended in a complete medium) were seeded in 50 μL drops on the inner surface of a multi-well plate lid and cultured for a day. The drops were observed under an inverted microscope, and the non-aggregated cells were manually counted. Cells in at least four fields per sample were counted.

Cell adhesion assay

A 96-well culture plate was coated with type I collagen (10 μg/mL) and fibronectin (10 μg/mL) overnight at 4°C. After washing with PBS, the plates were blocked with 3% BSA at room temperature for 1 h. Suspended cells in serum-free media were added to each coated well. After incubation for 30 min at 37°C, non-adherent cells were removed by streaming PBS over the plate three times. The remaining adherent cells were stained with 0.2% crystal violet and washed with PBS several times. The stained cells were dissolved in DMSO and the absorbance was measured at 570 nm.

Table S1. Genetic alterations in NSCLC cell lines used in this study.

Histology[1]	Name	Genetic alterations[2]						
		EGFR	*KRAS*	*TP53*	*STK11*	*BRAF*	*PTEN*	*PIK3CA*
ADC	Calu-1	WT	Mut	Null	WT	WT	WT	WT
ADC	H1975	Mut	WT	Mut	WT	WT	WT	Mut
ADC	HCC827	Mut	WT	Mut	WT	WT	WT	WT
ADC	H1944	WT	Mut	WT	Mut	WT	WT	WT
ADC	H292	WT	WT[3]	WT	WT	WT	WT	WT
ADC	H522	WT	WT	Mut	WT	WT	WT	WT
ADC	PC-9	Mut	WT	Mut	WT	WT	WT	WT
ADSQC	A549	WT	Mut	WT	Mut	WT	WT	WT
SQCC	H226B	WT	WT	WT	Un	Un	WT	Un
SQCC	H226Br	WT	Mut	Mut	Un	Un	Un	Un
SQCC	HCC15	WT	WT	Mut	Del	WT	WT	WT
LCC	H1299	WT	WT	Null	WT	WT	Null[4]	WT
LCC	H460	WT	Mut	WT	Mut	WT	WT	Mut

[1]ADC: Adenocarcinoma; ADSQC: Adenosquamous carcinoma; SQCC: Squamous cell carcinoma; LCC: large cell carcinoma

[2]Genetic alterations were retrieved from the Catalogue of Somatic Mutations in Cancer (COSMIC) database, the Cancer Cell Line Encyclopedia (CCLE) database, and literatures. WT: wild type; Mut: mutated; Un: unknown; Del: deletion; ND: not determined.

[3]KRAS G12S mutation has been identified in a previous study, but this mutation might be an artifact caused by a long-term culture and should be further validated[5].

[4]Undetectable expression caused by promoter methylation[6]

Table S2. Antibodies used in this study.

Target	Vendor	Catalogue Number	Clone	Dilution ratio	Application
Ninjurin1 (human)	Provided by Dr. Shin YK lab (SNU)		D12	1:100 (IF)<br>1 :1000 (IP)<br>1 μg /10^5 cells (FACS)	IF, IP, FACS
Ninjurin1 (mouse)	Provided by Dr. Kim KW lab (SNU)	MAB5105		1:100	IHC
Ninjurin1 (human)	R&D system	MAB5105	758943	1:1000	WB
Ninjurin1 (human)	R&D system	MAB51051	758926	1:100	IF
Nanog	Cell Signaling	4903S	D73G4	1:1000 (WB)<br>1:100 (IF)	WB, IF
Sox2	Abcam	ab97959		1:1000	WB
Oct4	Abcam	ab19857		1:1000	WB
Actin	Santa Cruz	sc-47778	C4	1:1000	WB
GAPDH	Cell Signaling	5174	D16H11	1:1000	WB
OctA-probe	Santa Cruz	sc-166355	H-5	1:1000 (WB)<br>1:100 (IF)<br>1:1000 (IP)	WB, IF, IP
CC10	Santa Cruz	sc-9772	T-18	1:100	IF
Prosurfactant protein C	Merck	AB3786		1:100	IF
β-catenin	Santa Cruz	sc-7963	E-5	1:1,000 (WB)<br>1:100 (IF)	WB, IF
Dvl3	Cell Signaling	3218		1:1,000	WB
Actin	Santa Cruz	sc-47778	C4	1:1,000	WB
Cleaved PARP (Cl-PARP)	BD	552596	F21-852	1:1,000	WB
PARP	Cell Signaling	9542		1:1,000	WB

Cleaved caspase-3 (Cl-Cas3)	Cell Signaling	9661S	D175	1:1000	WB
β-tubulin	Cell Signaling	2128	9F3	1:1,000	WB
His	Santa Cruz	sc-8036	H-3	1:1,000	WB
GST	Santa Cruz	sc-459	Z-5	1:1,000	WB
LRP6	Cell Signaling	2560	C5C7	1:1,000	WB
LRP6	Santa Cruz	sc-25317	C-10	1:1000	IP
phospho-LRP6 (pLRP6, S1490)	Cell Signaling	2568		1:1,000	WB
FZD2	LSBio	LS-C166295		1:1,000	WB
Axin1	Cell Signaling	2087	C76H11	1:1,000	WB
GSK3β	Cell Signaling	9832	3D10	1:1,000	WB
HRP-conjugated goat anti-mouse IgG	GeneTex	213111-01		1:5,000	WB
HRP-conjugated goat anti-rabbit IgG	GeneTex	213110-01		1:5,000	WB
Alexa Fluor 488-conjugated goat anti-rabbit IgG	Thermo Fisher Scientific	A-11034		1:1,000	IF
Alexa Fluor 488-conjugated goat anti-mouse IgG	Thermo Fisher Scientific	A11001		1:1,000	IF
Alexa Fluor 594-conjugated goat anti-rabbit IgG	Thermo Fisher Scientific	A11012		1:1000	IF
Alexa Fluor 594-conjugated donkey anti-mouse igG	Thermo Fisher Scientific	A21203		1:1000	IF

FITC-conjugated Donkey anti-Human IgG	Jackson ImmunoResearch	709-095-098	1:1,000	IF
Goat anti-Rabbit IgG (H+L) Biotinylated	Bethyl	A120-101B	1:1,000	IHC

[1)]Application - WB: Western blot analysis; IF: Immunofluorescence staining; IHC: Immunohistochemistry; IP: Immunoprecipitation; FACS: Fluorescence-Activated Cell Sorting

Table S3. Primer sequences used in this study.

Gene	Forward sequence (5´-3´)	Reverse sequence (5´-3´)	Application
NINJ1	CTCGACCCACTCAAGGACAC	GTAGGGCCAAAGGCAGTTCT	Real-time PCR
ALDH1A1	CGGGAAAAGCAATCTGAAGAGGG	GATGCGGCTATACAACACTGGC	Real-time PCR
POU5F1	CTGGGTTGATCCTCGGACCT	CCATCGGAGTTGCTCTCCA	Real-time PCR
NANOG	TTTGTGGGCCTGAAGAAAACT	AGGGCTGTCCTGAATAAGCAG	Real-time PCR
SOX2	GCCGAGTGGAAACTTTTGTCG	GGCAGCGTGTACTTATCCTTCT	Real-time PCR
MYC	CAGCTGCTTAGACGCTGGATT	GTAGAAATACGGCTGCACCGA	Real-time PCR
AXIN2	AGTGTGAGGTCCACGGAAAC	CTTCACACTGCGATGCATTT	Real-time PCR
GLI1	AGCCTTCAGCAATGCCAGTGAC	GTCAGGACCATGCACTGTCTTG	Real-time PCR
GLI2	GTCAGAGCCATCAAGACCGAGA	GCATCTCCACGCCACTGTCATT	Real-time PCR
HES1	TCAACACGACACCGGATAAAC	GCCGCGAGCTATCTTTCTTCA	Real-time PCR
HEY1	GTTCGGCTCTAGGTTCCATGT	CGTCGGCGCTTCTCAATTATTC	Real-time PCR
HEY2	AAGGCGTCGGGATCGGATAA	AGAGCGTGTGCGTCAAAGTAG	Real-time PCR
CTGF	CTTGCGAAGCTGACCTGGAAGA	CCGTCGGTACATACTCCACAGA	Real-time PCR
AXL	GTTTGGAGCTGTGATGGAAGGC	CGCTTCACTCAGGAAATCCTCC	Real-time PCR
CYR61	GGAAAAGGCAGCTCACTGAAGC	GGAGATACCAGTTCCACAGGTC	Real-time PCR
LRP5	AGACCGTACAGGCCCTACAT	TAGTCGCTGTCACACACGTC	Real-time PCR
LRP6	AAACAGACGGGACTTGCGAT	AAACACAAAGTCCACCGCAG	Real-time PCR
DVL2	CTGGAGCCTGAGACAGAAACC	CATGCTCACTGCTGTCTCTCCT	Real-time PCR
DVL3	GCAGCGACCCAGCTATAAGT	TGAAGCATGGTAGCTTGGCA	Real-time PCR
WNT1	CTCTTCGGCAAGATCGTCAACC	CGATGGAACCTTCTGAGCAGGA	Real-time PCR
WNT2	AGGATGCCAGAGCCCTGATGAA	AGCCAGCATGTCCTGAGAGTAC	Real-time PCR
WNT3	GCGTGTTAGTGTCCAGGGAGTT	TGAGGTGCATGTGGTCCAGGAT	Real-time PCR
WNT3A	ATGAACCGCCACAACAACGAGG	GTCCTTGAGGAAGTCACCGATG	Real-time PCR
WNT4	GCTGGAGAAGTGCGGCTGTGA	CCACAAACGACTGTGAGAAGGC	Real-time PCR
WNT5A	TACGAGAGTGCTCGCATCCTCA	TGTCTTCAGGCTACATGAGCCG	Real-time PCR
WNT6	TGGCCTCTAGGAGGAAACAGT	ATTGATACTAACCTCACCCACC	Real-time PCR

WNT7A	AGGAGAAGGCTCACAAATGGGC	CGGCAATGATGGCGTAGGTGAA	Real-time PCR
WMT8A	AGGCATGTGACCATGTCCA	ATTTGTAGCCACAGGCTGCT	Real-time PCR
FZD1	GGGGCTTAACAACGTGGAC	CAGAAAGGACGTGCCGATAAA	Real-time PCR
FZD2	GTGCCATCCTATCTCAGCTACA	CTGCATGTCTACCAAGTACGTG	Real-time PCR
FZD3	GGCTCTCATAGTTGGCATTCCC	TGGAGTACCTGTCGGCTCTCAT	Real-time PCR
FZD4	CCTCGGCTACAACGTGACC	TGCACATTGGCACATAAACAGA	Real-time PCR
FZD5	CCGTTCGTGTGCAAGTGTC	GAAGCGTTCCATGTCGATGAG	Real-time PCR
FZD6	GGCAGTGTATCTGAAAGTGCGC	GATGTGGAACCTTTGAGGCTGC	Real-time PCR
FZD7	GTCTTCAGCGTGCTCTACACAG	ACGGCATAGCTCTTGCACGTCT	Real-time PCR
FZD8	GCTCTACAACCGCGTCAAGACA	AAGGTGGACACGAAGCAGAGCA	Real-time PCR
FZD9	GTTCCAGTACGTGGAGAAGAGC	CAGCAAGAAGGTGAGCACAGTG	Real-time PCR
FZD10	GAACACGGACAAGCTGGAGAAG	GGCGTTCGTAAAAGTAGCAGGC	Real-time PCR
ACTB	GCGAGAAGATGACCCAGATC	GGATAGCACAGCCTGGATAG	Real-time PCR
LRP5	GGACACCAACATGATCGAGTCG	CGCTCAATGCTGTGCAGATTCC	RT-PCR
LRP6	CAGTTGGAGTGGTGCTGAAAGG	CCATCCAAAGCAGCCCGTTCAA	RT-PCR
DVL2	CAGGATTTCGGGGTGGTGAA	AGCGCCATGCTCACTGC	RT-PCR
DVL3	GTCGTGTACTGGCTGGTGTC	TCCATTTAGCCGGGTTGCAT	RT-PCR
WNT1	CAAGATCGTCAACCGAGGCT	AAGGTTCATGAGGAAGCGCA	RT-PCR
WNT2	CTGGGGCGGCCTTACAATAA	AGGCAGCCTCTCCATTTTCC	RT-PCR
WNT3	CTGGCTACCCAATTTGGTGGT	CATCTATGGTGGTGCAGTTCCA	RT-PCR
WNT3A	AAGCAGGCTCTGGGCAGCTA	GACGGTGGTGCAGTTCCA	RT-PCR
WNT4	GAGCAACTGGCTGTACCTGG	TGCCGAAGAGATGGCGTACA	RT-PCR
WNT5A	CCTCCATTCCTGGGCGCAT	GGACTTCTTCATGGCGAGGG	RT-PCR
WNT6	CGTAGGGCGGTCACGATG	AACTGGAACTGGCACTCTCG	RT-PCR
WNT7A	ACTTAGGGGTAAGGAGGGGC	TGAGCTTAACAGGCTGAGGC	RT-PCR
WMT8A	CCTTCCTTCCTAGCGCAGAG	TTGGACATGGTCACATGCCT	RT-PCR
FZD1	CTCTACTTCTTCAGCATGGCCA	TCCACGTTGTTAAGCCCCA	RT-PCR

FZD2	CCATCCTATCTCAGCTACAAGTTTCT	GCAGCCCTCCTTCTTGGT	RT-PCR
FZD3	TCCCCTCTGCCTGTATGTGGTAGT	GCTGCTCACTTTGCTGTGGA	RT-PCR
FZD4	CTCGGCTACAACGTGACCAAGAT	AATATGATGGGGCGCTCAGGGTA	RT-PCR
FZD5	GTGCCCATTCTGAAGGAGTCAC	TCCATGTCGATGAGGAAGGTG	RT-PCR
FZD6	ACTCTTGCCACTGTGCCTTTG	GTCGAGCTTTTGCTTTTGCCT	RT-PCR
FZD7	CAAGACCGAGAAGCTGGAGAAG	TGCCGACGATCATGGTCAT	RT-PCR
FZD8	GGACTACAACCGCACCGACCT	ACCACAGGCCGATCCAGAAGAC	RT-PCR
FZD9	TCAAGGTCAGGCAAGTGAGCA	AGCTTCCAGAGGAACGCAACA	RT-PCR
FZD10	CAGGTGTGCAGCCGTAGGTTAA	AAGCACCACATCTTAGCTCCGG	RT-PCR
ACTB	ACTACCTCATGAAGATC	GATCCACATCTGCTGGAA	RT-PCR
Ninj1	CAAGCTTGATCCAGTACTAGTGAAC	CGCTCTTCTTGTTGGCATAATGGTT	RT-PCR
Actb	CATTGCTGACAGGATGCAGAAGG	TGCTGGAAGGTGGACAGTGAGG	RT-PCR

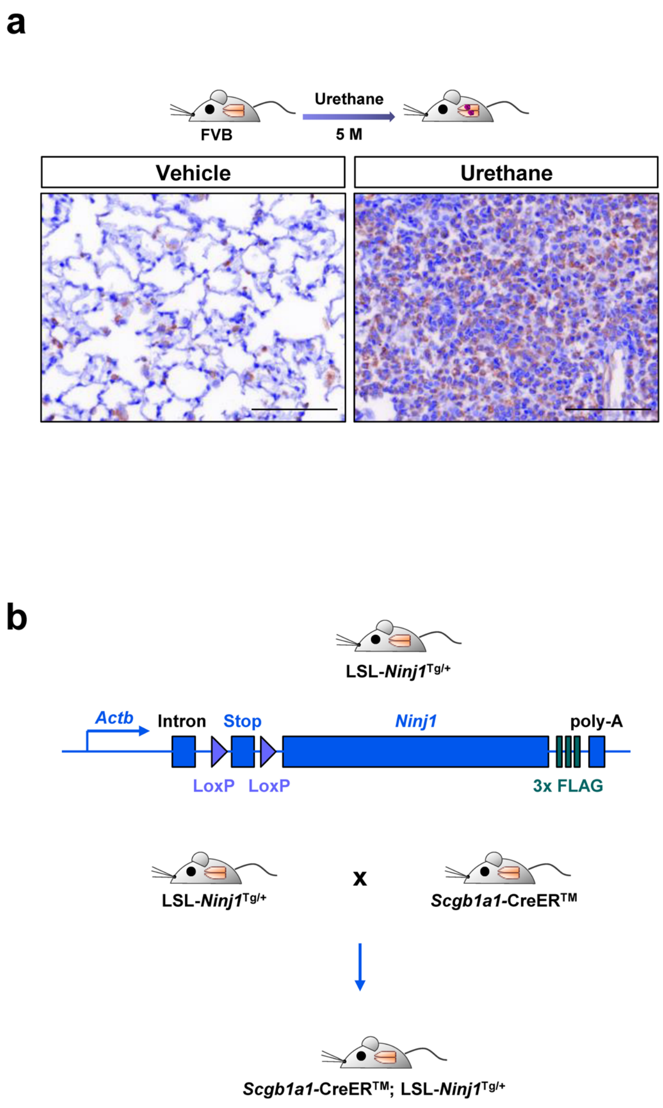

Fig. S1. Increased expression of Ninj1 in lung tumor in response to urethane treatment, schematic diagram for generating the conditional *Ninj1* overexpression in mice using the Cre-LoxP system, and breeding strategies for the establishment of mouse models. (**a**) Immunohistochemical analysis of Ninj1 expression in normal lung tissues from vehicle-treated mice and lung tumor tissues from urethane-treated mice. Scale bars: 100 μm. (**b**) The construct for LoxP-Stop-LoxP (LSL)-*Ninj1*$^{Tg/+}$ for the transgenic (Tg) mouse model harboring conditional *Ninj1* overexpression using the Cre-LoxP system. A breeding strategy between LSL-*Ninj1*$^{Tg/+}$ and *Scgb1a1*-CreER^{TM} mice to obtain *Scgb1a1*-CreER^{TM};LSL-*Ninj1*$^{Tg/+}$ mice. Ninj1: Ninjurin1.

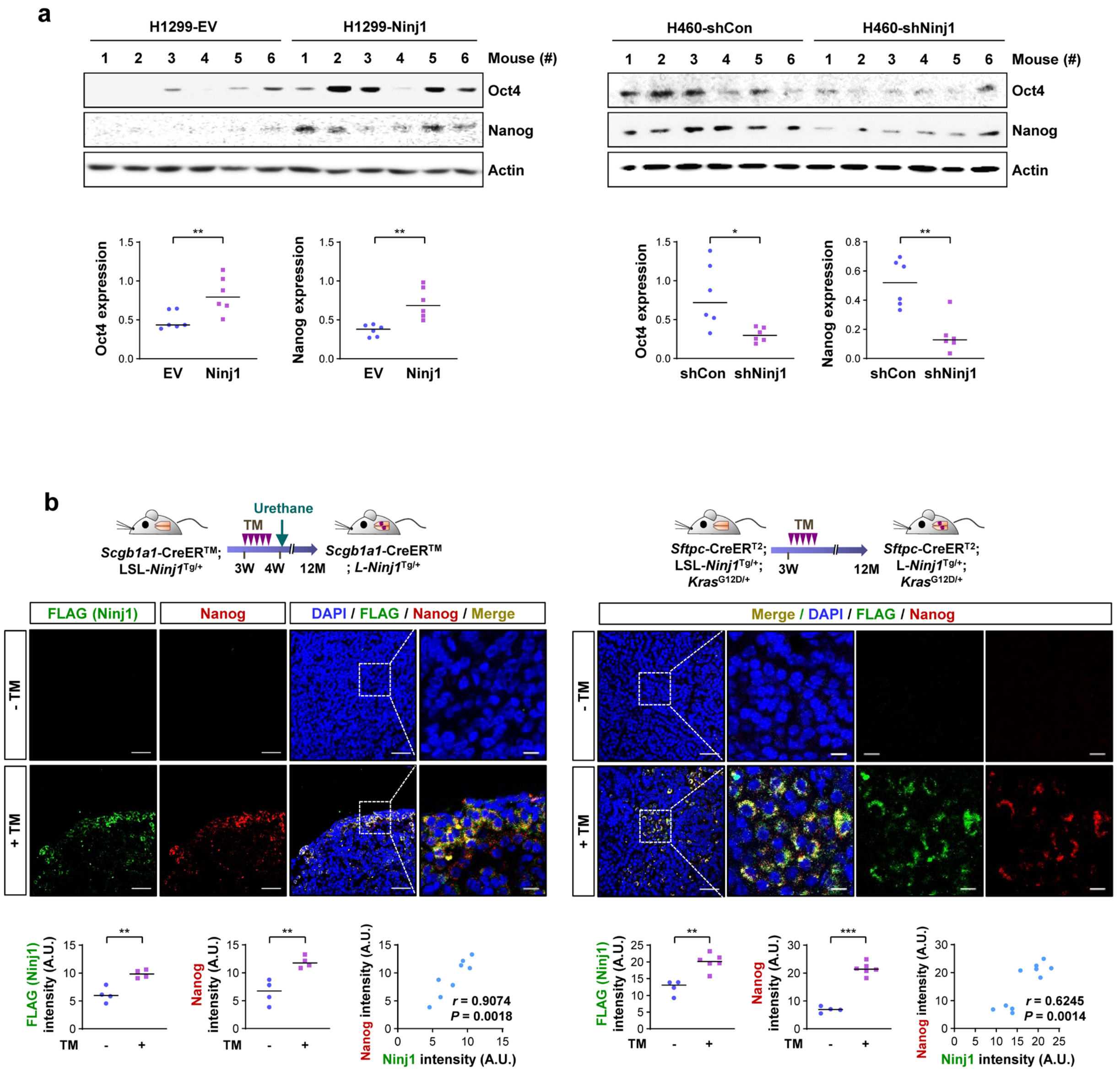

Fig. S2. Association of Ninj1 with Oct4 or Nanog protein expression in xenograft tumors from NSCLC cells and the regulation of Nanog expression by tamoxifen-mediated Ninj1 induction in mice. (**a**) Western blot analysis examining Oct4 and Nanog protein expression in xenograft tumors of H1299-Ninj1 (**left**) or H460-shNinj1 cells (**right**) and in those of their control tumors. Densitometric analysis was performed using ImageJ software. (**b**) Immunofluorescence analysis examining the lungs from *Scgb1a1*-CreER^{TM};LSL-Ninj1$^{Tg/+}$ mice (**left**) and *Sftpc*-CreER^{T2};LSL-Ninj1$^{Tg/+}$;*Kras*$^{G12D/+}$ mice (**right**) for correlation of NInj1 and Nanog expression levels. The Ninj1^{+} cells were determined using ImageJ software. Scale bars: 20 μm. The significance of the correlation was determined using the Pearson correlation test. Statistical significance of the difference between two groups was determined by a two-tailed Student's t-test or Mann-Whitney test. Ninj1: Ninjurin1. TM: tamoxifen.

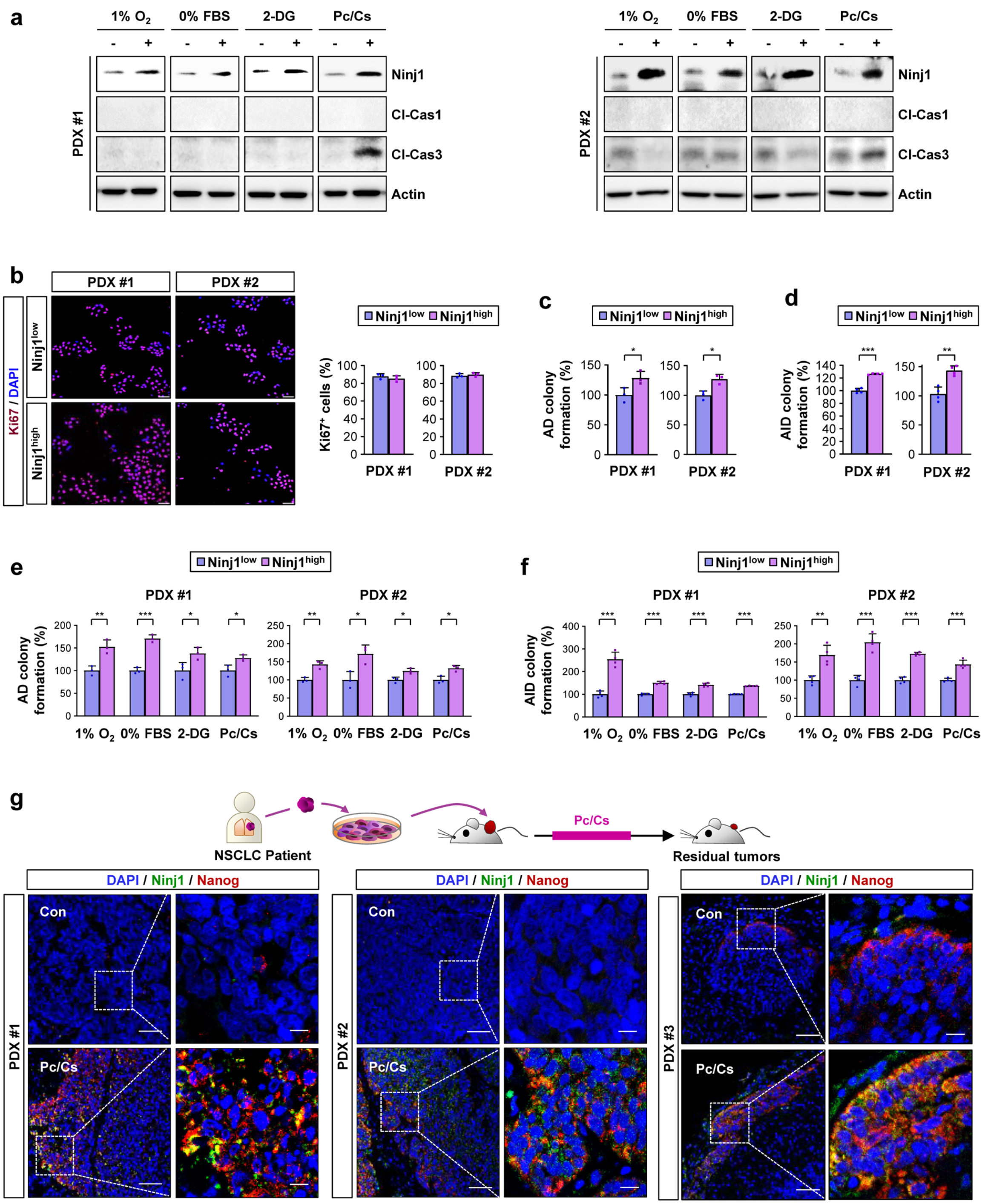

Fig. S3. Induction of Ninj1 expression in hazardous environments and increased survival capacity in Ninj1^{high} populations. (**a**) Western blot analysis examining Ninj1, cleaved caspase 1 (Cl-Cas1), and cleaved caspase 3 (Cl-Cas3) in primary cultured patient-derived xenograft (PDX)-derived tumor cells under culture conditions of hypoxia (1% O_2), serum starvation (0% FBS), glucose deprivation (1 mM 2-deoxy-L-glucose [2-DG]), and chemotherapy treatment (10 nM

paclitaxel and 10 μM cisplatin in combination; Pc/Cs). (**b-f**) The basal Ki67 positivity (**b**), anchorage-dependent (AD) (**c**) and -independent (AID) (**d**) colony formation, and anchorage-dependent (**e**) and -independent (**f**) colony formation under hypoxia (1% O_2), serum starvation, glucose deprivation, and exposure to chemotherapy (Pc/Cs) in the $Ninj1^{low}$ and $Ninj1^{high}$ populations of two PDX-derived primary cultured cancer cells. Scale bars: 50 μm (**b**). (**g**) Representative images of IF analyses examining the levels of Ninj1 and Nanog expression in three PDX tumors treated with chemotherapy (paclitaxel and cisplatin in combination; Pc/Cs). The quantification analysis results for the IF images are included in **Fig. 4l**. Scale bars: 50 μm. Scale bars (inset): 10 μm. The bars represent the mean ± SD; **P* < 0.05, ***P* < 0.01, and ****P* < 0.001, as determined by a two-tailed Student's t-test by comparison to the indicated group. Ninj1: Ninjurin1.

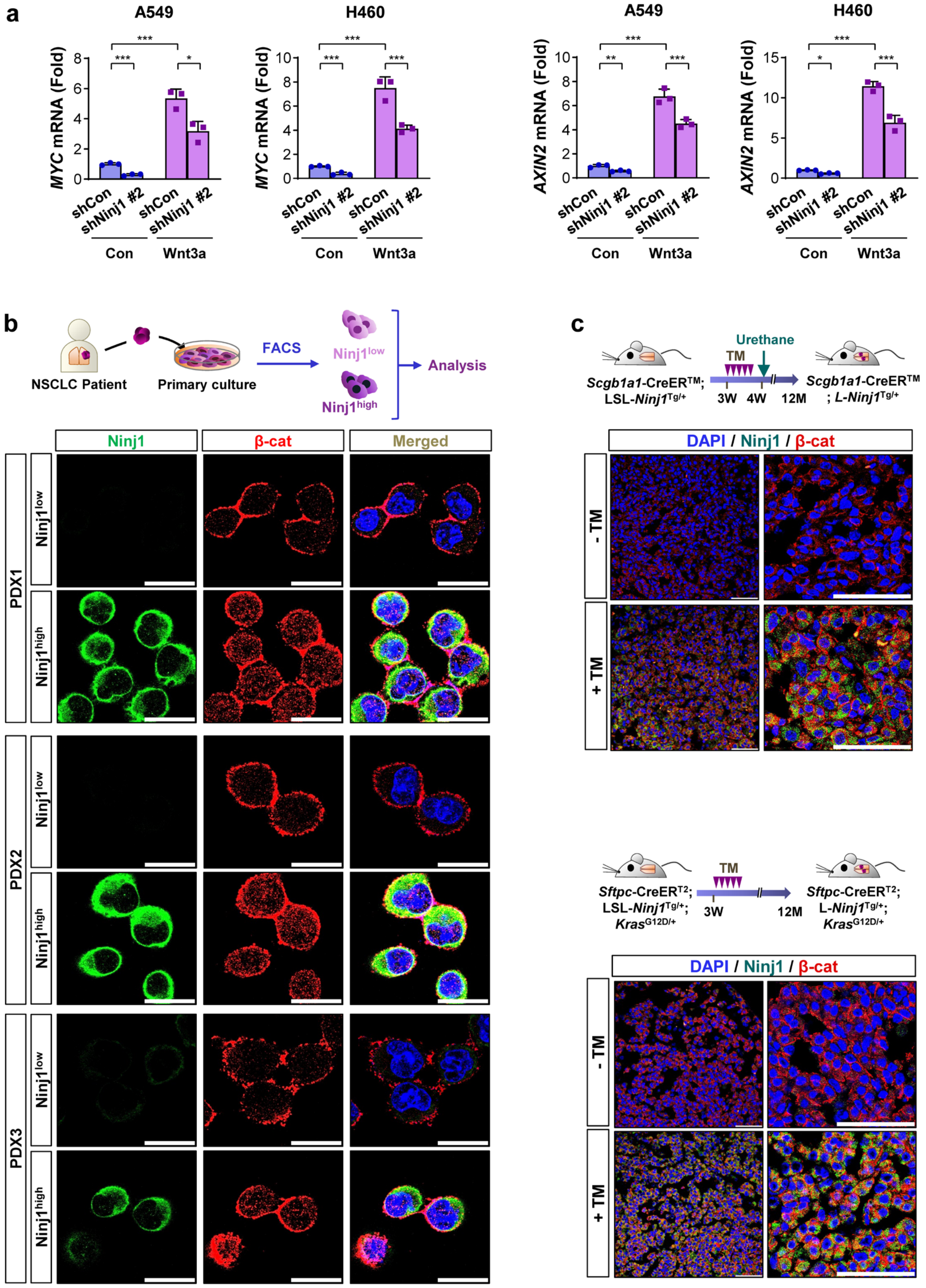

Fig. S4. Role of Ninj1 in the activation of the Wnt/β-catenin signaling pathway. (**a**) Real-time PCR analysis examining *MYC* and *AXIN2* in the indicated stable NSCLC cells with knockdown of Ninj1 expression in the absence or presence of Wnt3a conditioned medium (Wnt3a). The bars represent the mean ± SD; **P* < 0.05, ***P* < 0.01, and ****P* < 0.001, as determined by one-way ANOVA with Tukey's post-hoc test. (**b, c**) Representative immunofluorescence analysis images

revealing the levels of Ninj1 and β-catenin (β-cat) expression in FACS-sorted Ninj1^{high} and Ninj1^{low} populations of three patient-derived xenograft (PDX)-derived primary cultured cancer cells (**b**), CCSP^{+} club cells (**c, top**) and SPC^{+} type II alveolar epithelial cells (**c, bottom**). The quantification analysis results for the IF images are included in **Fig. 5h and 5i**. Scale bars: 50 μm. Ninj1: Ninjurin1.

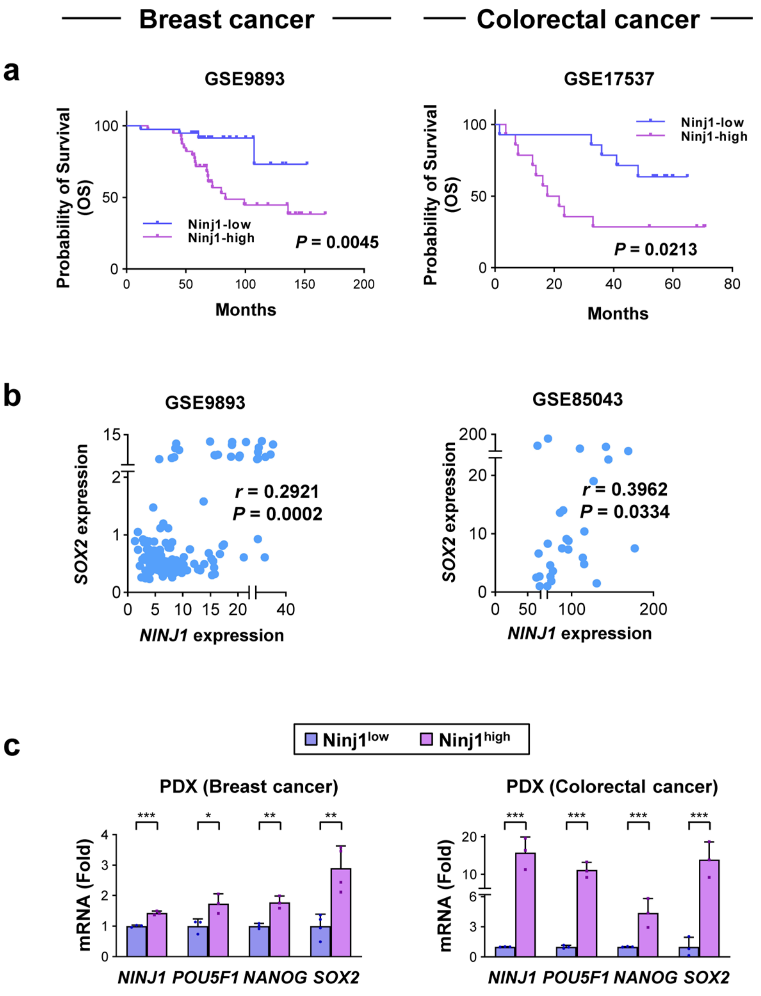

Fig. S5. Analysis of prognostic impact of Ninj1 expression in patients with breast or colorectal cancers and the association of Ninj1 expression levels with CSC marker gene expression in patient-derived tumors. (**a**) The Kaplan–Meier estimates for the association of *NINJ1* expression with overall survival (OS) of patients with breast and colorectal cancers. The *P* value was determined using the log-rank test. (**b**) Analysis of GEO datasets for the relationship between *NINJ1* and *SOX2* expression in breast and colorectal cancers. The significance of the correlation was determined using the Spearman rank correlation test. (**c**) Real-time PCR examining changes in the mRNA expression of *NINJ1* and CSC markers (*POU5F1*, *NANOG*, and *SOX2*) in the $Ninj1^{low}$ and $Ninj1^{high}$ populations of patient-derived xenograft (PDX)-derived primary breast and colon tumor cells. The bars represent the mean ± SD; **P* < 0.05, ***P* < 0.01, and ****P* < 0.001, as determined by a two-tailed Student's *t*-test by comparison to the indicated group.

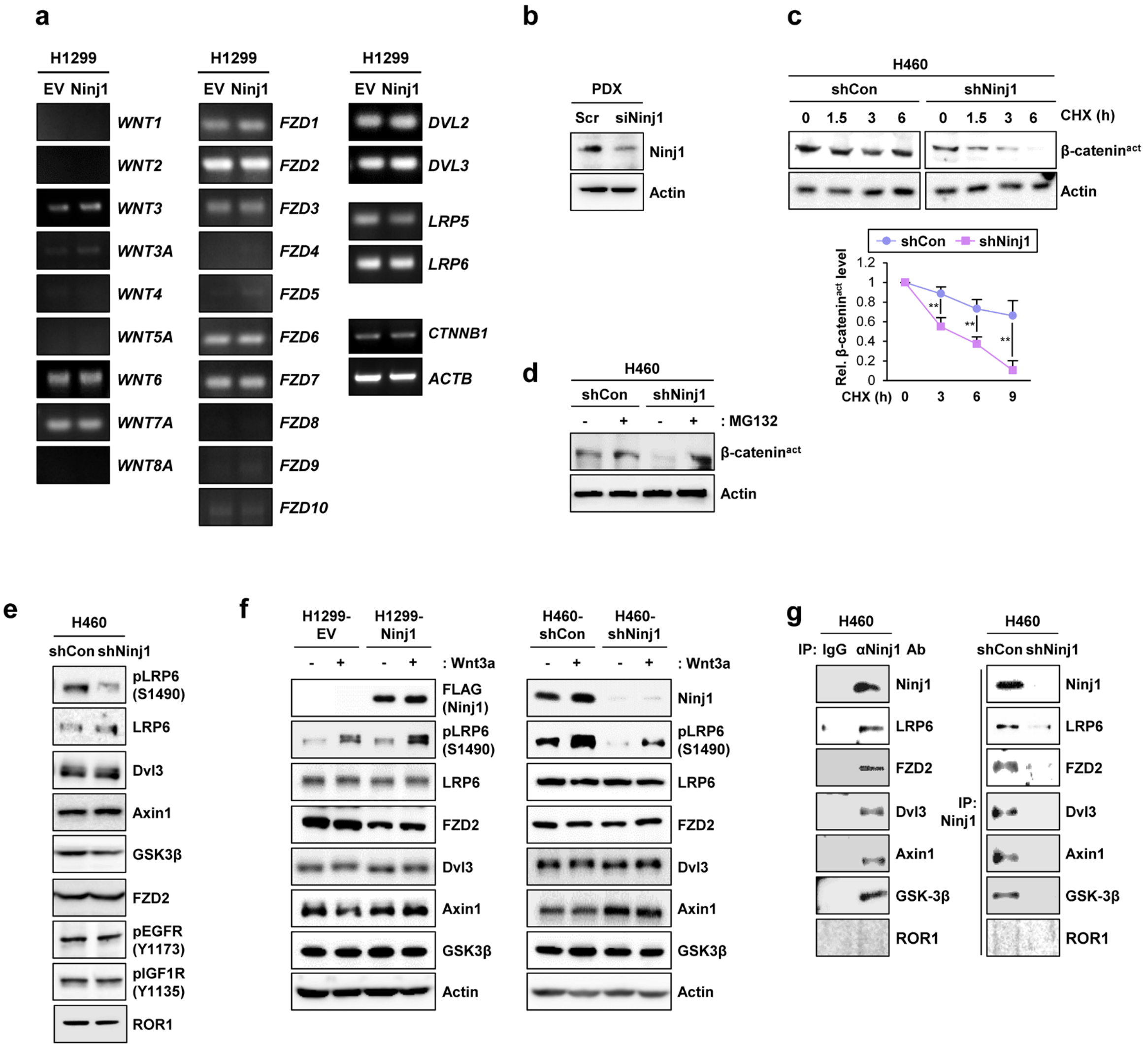

Fig. S6. mRNA and protein expression levels of the Wnt/β-catenin signaling components and the role of Ninjurin1 in β-catenin protein stability, activation of the Wnt signaling pathway, and formation of the LRP6-FZD2 signalosome. (**a**) RT-PCR analysis examining mRNA expression of Wnt ligands (WNT1-WNT8A), Frizzled receptors (FZD1-FZD10), LRP (LRP5 and LRP6), DVL (DVL2 and DVL3), and β-catenin (*CTNNB1*) in H1299-EV and H1299-Ninj1 cells. (**b**) Western blot (WB) analysis examining the regulation of Ninj1 expression in primary patient-derived xenograft tumor cells transfected with Ninj1 siRNAs. (**c**, **d**) WB analysis examining active β-catenin (β-catenin^{act}) protein in H460-shCon and H460-shNinj1 cells after treatment with cycloheximide (CHX, 100 μg/mL) for the indicated time-points (**c**) or after treatment with MG132 (10 μM) (**d**). The bars represent the mean ± SD; $^{**}P < 0.01$, as determined by a two-tailed Student’s t-test. (**e**) WB analysis examining the expression of the indicated canonical Wnt/β-catenin signaling components and the phosphorylation of EGFR, IGF-1R, and ROR1 in H460-shCon and H460-shNinj1 cells. (**f**) WB analysis examining the expression of the indicated canonical Wnt/β-catenin signaling components in H460-shNinj1, H1299-Ninj1, and their control

cells in the absence or presence of Wnt3a conditioned medium (Wnt3a). (**g**) WB analysis of IgG and anti-Ninj1 immunoprecipitates examining the indicated Wnt/β-catenin signaling proteins to determine interactions among Ninj1, LRP6, FZD2, and other Wnt/β-catenin signaling components in H460-shCon and H460-shNinj1 cells.

References

1. Livak KJ, Schmittgen TD: **Analysis of relative gene expression data using real-time quantitative PCR and the 2(T)(-Delta Delta C) method.** *Methods* 2001, **25:**402-408.

2. Shin MW, Bae SJ, Wee HJ, Lee HJ, Ahn BJ, Le H, Lee EJ, Kim RH, Lee HS, Seo JH, et al: **Ninjurin1 regulates lipopolysaccharide-induced inflammation through direct binding.** *Int J Oncol* 2016, **48:**821-828.

3. Oh SH, Woo JK, Yazici YD, Myers JN, Kim WY, Jin Q, Hong SS, Park HJ, Suh YG, Kim KW, et al: **Structural basis for depletion of heat shock protein 90 client proteins by deguelin.** *J Natl Cancer Inst* 2007, **99:**949-961.

4. Hu Y, Smyth GK: **ELDA: extreme limiting dilution analysis for comparing depleted and enriched populations in stem cell and other assays.** *J Immunol Methods* 2009, **347:**70-78.

5. Deschoolmeester V, Boeckx C, Baay M, Weyler J, Wuyts W, Van Marck E, Peeters M, Lardon F, Vermorken JB: **KRAS mutation detection and prognostic potential in sporadic colorectal cancer using high-resolution melting analysis.** *Br J Cancer* 2010, **103:**1627-1636.

6. Soria JC, Lee HY, Lee JI, Wang L, Issa JP, Kemp BL, Liu DD, Kurie JM, Mao L, Khuri FR: **Lack of PTEN expression in non-small cell lung cancer could be related to promoter methylation.** *Clin Cancer Res* 2002, **8:**1178-1184.
